# Mechanism for exercise-mediated prevention against muscle wasting on extensor digitorum longus muscle in Spontaneously Diabetic Torii fatty rats

**DOI:** 10.1186/s12576-023-00865-5

**Published:** 2023-04-04

**Authors:** Hitoshi Kotake, Yuji Ogura, Shohei Yamada, Kazuho Inoue, Shiika Watanabe, Daisuke Ichikawa, Takeshi Sugaya, Keiichi Ohata, Yasunori Natsuki, Seiko Hoshino, Minoru Watanabe, Kenjiro Kimura, Yugo Shibagaki, Atsuko Kamijo-Ikemori

**Affiliations:** 1 Division of Nephrology and Hypertension, Department of Internal Medicine, St. Marianna University School of Medicine, Kanagawa, Japan; 2 Department of Physiology, St. Marianna University School of Medicine, Kanagawa, Japan; 3 Department of Anatomy, St. Marianna University School of Medicine, Miyamae-Ku, Kawasaki, Kanagawa 216-8511 Japan; 4 Institute for Ultrastructural Morphology, St. Marianna University School of Medicine, Kawasaki, Japan; 5 Institute for Animal Experimentation, St. Marianna University Graduate School of Medicine, Kanagawa, Japan; 6 JCHO Tokyo Takanawa Hospital, Tokyo, Japan

**Keywords:** Type 2 diabetes, Exercise, Muscle, Extensor digitorum longus muscle, Type IIb muscle fibers, Angiogenic response

## Abstract

We previously reported the significant increase in limb muscle strength and cross-sectional area of the type IIb muscle fibers in the extensor digitorum longus (EDL) muscle in a type 2 diabetic animal model, with Spontaneously Diabetic Torii (SDT) fatty rats (n = 6) undergoing regular treadmill exercise from 8 to 16 weeks of age compared with sedentary SDT fatty rats (n = 6). This study investigated the mechanism by which exercise training prevented skeletal muscle wasting in the EDL muscle of the SDT fatty rats. The endurance exercise for 8 weeks downregulated the expression of muscle RING-finger protein-1 (an E3 ubiquitin ligase) and upregulated the expression of CD31, insulin receptor substrate-2, and phosphorylated endothelial nitric oxide synthase in the EDL muscle of 16-week-old SDT fatty rats.

Endurance exercise training might reduce muscle wasting by preventing muscle degradation and increasing the angiogenic response in the EDL muscle in type 2 diabetes.

**Supplementary Information:**

The online version contains supplementary material available at 10.1186/s12576-023-00865-5.

## Background

Muscle wasting prevention has been a major focus in our aging society, given the increasing evidence that muscle wasting is associated with increased mortality rates [1, 2]. In diabetes in particular, which presents abnormal glucose metabolism, there is a high risk of muscle wasting [3–5]. Managing diabetes is therefore clinically important for promoting the health and well-being of patients with diabetes by preventing muscle wasting and improving glucose metabolism [6].

Exercise has been reported to prevent muscle wasting in patients with diabetes [7, 8]. Studies have reported the underlying mechanisms by which exercise prevents muscle wasting in diabetes, such as the activation of muscle protein synthesis via the upregulated expression of insulin-like growth factor-1 (IGF-1) [9], prevention of muscle protein degradation due to restored mitochondrial function via the activation of 5′-adenosine monophosphate-activated protein kinase (AMPK) [10] or due to oxidative stress [11], and retention of a certain level of oxidative stress [12]. Exercise has also been related to accelerated angiogenesis [13, 14], which maintains muscle mass via the increased production of adenosine triphosphate in the mitochondria through the increased oxygen supply to the muscle.

Diabetes has numerous comorbidities, such as hypertension, hyperlipidemia, and chronic kidney disease (CKD), which are known to be associated with accelerated muscle wasting [15–17]. However, the effectiveness of exercise and mechanisms by which it prevents muscle weakness in patients with diabetes and multiple complications have not been fully investigated. We previously reported that male Spontaneously Diabetic Torii (SDT) fatty rats (SDT.Cg-*Lepr*^fa^/JttJcl), as a type 2 diabetic animal model, presented hyperglycemia, hypertension, hyperlipidemia, and CKD and that regular treadmill exercise improved their limb muscle strength and the cross-sectional area of the type IIb muscle fibers in their extensor digitorum longus (EDL) muscle (Additional file 1: Table. S1–3) [18]. Therefore, the purpose of this study is to investigate the mechanism by which exercise training prevents skeletal muscle weakness in the EDL of SDT fatty rats.

## Materials and methods

### Animals

The SDT fatty rat was established by introducing the *lepr(fa)* allele of the Zucker fatty rat into the genome of a nonobese SDT rat, which represents an inbred strain of Sprague-Dawley (SD) rat [19]. We previously used 5-week-old male SDT fatty rats (n = 12) and age-matched control male SD rats (n = 10) (CLEA Japan, Tokyo, Japan) and reported that regular treadmill exercise was associated with increased muscle strength and cross-sectional area of the type IIb muscle fibers in EDL (Additional file 1: Table S1-3), as well as attenuation of hypertension, hyperlipidemia, and CKD in male SDT fatty rats [18]. This study used the EDL and soleus muscles removed when the leg muscle tissues and kidneys were evaluated in the previous study [18], and analyzed the mechanism by which exercise attenuates muscle weakness.

In the previous study, leg muscle tissue was removed under general anesthesia using the inhaled anesthetic 2% isoflurane 96 h after finishing the exercise protocol [18]. The extracted muscle specimens were categorized as fast (EDL) or slow (soleus). After weighing, each muscle was individually frozen in liquid nitrogen and stored at −80°C until further analysis.

The experiments were combined to ensure the efficient use of experimental animals and to adhere to the “Reduction” part of the “3Rs” (Replacement, Reduction, and Refinement) provided in the Act on the Welfare and Management of Animals. All procedures performed in the studies involving animals were in accordance with the ethical standards of the St. Marianna University School of Medicine (Japan) or of the institution in which the studies were conducted (Approval Number: 2008012). The use of the rats was approved by the appropriate institutional review committee at the St. Marianna University School of Medicine.

### Exercise protocol

In the previous experimental protocol, regular treadmill exercise was performed from the age of 8 to 16 weeks on the SDT fatty rats (n = 6) and SD rats (n = 5) [18]. The sedentary animals (six SDT fatty rats and five SD rats) were compared with the exercise animals. The regular treadmill exercise was performed on a motor-driven treadmill (KN-73 TREAD-MILL; Natume Seisakusho, Co., Ltd., Tokyo, Japan), 4 times a week from ages 8 to 16 weeks. Exercise intensity was described in detail in our previous study [18]. After a warm-up with a slowly increasing speed from 5 to 10 m/min for 5 min, the prescribed exercise with a 3° slope was started at 10 m/min for 10 min/day for 4 times a week for the 1st week. From the second to the 8th week, the speed and duration of the prescribed exercise were gradually increased to 16–20 m/min, depending on the rats’ tolerance, for 30 min after the 5-min warm-up for 4 times a week.

### Real-time quantitative reverse transcription polymerase chain reaction

Total RNA was extracted from these EDL and soleus muscles using an RNeasy Midi kit (Qiagen, Valencia, CA, USA), and a TaqMan real-time polymerase chain reaction with a StepOnePlus real-time polymerase chain reaction system (Applied Biosystems, Waltham, MA, USA) was used to measure the mRNA levels of *muscle RING-finger protein-1 (MuRF1)* and *18S ribosomal RNA (18s*) to evaluate the protein degradation system. The expression of *MuRF1* mRNA levels was normalized to the levels of *18s* in all samples.

### Evaluation of insulin-like growth factor-1 (IGF-1) in the muscle

Proteins were extracted from the EDL and soleus muscles, and their concentrations were determined as previously described [20]. Given that IGF-1 was related to accelerated muscle growth [21], IGF-1 protein expression was measured using a rat IGF-1 immunoassay kit (R&D Systems, Inc., Minneapolis, MN, USA). The levels in the EDL and soleus muscles were corrected to the protein concentration of each sample.

### Western blot analysis

The protein (30 µg) extracted from the EDL and soleus muscles was separated by electrophoresis and subjected to immunoblotting. After blocking, the membranes were incubated overnight at 4 °C with primary antibodies against Akt (rabbit monoclonal; #9272; 1 : 1000; Cell Signaling Technology, Inc., Danvers, MA, USA), phospho-Akt (Ser473) (rabbit monoclonal; #9271; 1 : 1000; Cell Signaling Technology, Inc.), ribosomal protein S6 kinase beta-1 (S6K; rabbit monoclonal; #2708; 1:1000; Cell Signaling Technology), phospho-p70S6 kinase (S6K; rabbit monoclonal; #9234; 1:1000; Cell Signaling Technology), forkhead box protein O1 (FOXO1; rabbit monoclonal; #9454; 1:1000; Cell Signaling Technology), phospho- FOXO1 (Ser256; rabbit monoclonal; #9461; 1:1000; Cell Signaling Technology, Inc.), Nuclear Factor-κB p65 (NF-κB; rabbit monoclonal; #8242; 1:1000; Cell Signaling Technology), and phospho-NF-κB p65 (Ser536; rabbit monoclonal; #3033; 1:1000; Cell Signaling Technology), MuRF-1 (rabbit polyclonal; MP3401; 1:1000; ECM Biosciences, Versailles, KY, USA), AMPK (mouse monoclonal; No. 2793; 1:1000; Cell Signaling Technology), phospho-AMPK (Thr172; rabbit monoclonal; No. 2535; 1:1000; Cell Signaling Technology), proliferator-activated receptor gamma-coactivator-1 alpha (PGC1α; rabbit polyclonal; ab191838; 1:1000; Abcam Japan, Tokyo, Japan), CD31 (rabbit monoclonal; ab222783; 1:2000; Abcam), insulin receptor substrate 2 (IRS2; rabbit polyclonal; No. 4502; 1:1000; Cell Signaling Technology), endothelial nitric oxide synthase (eNOS; rabbit monoclonal; No. 32027; 1:1000; Cell Signaling Technology), and phospho-eNOS (Ser1177; rabbit monoclonal; MA5-14957; 1:1000; Thermo Fisher Scientific, Rockford, IL, USA). We also used a rabbit monoclonal antibody to α-tubulin (ab176560; 1:4000-8000, Abcam) to detect α-tubulin on the same membranes. After incubation with horseradish peroxidase-conjugated anti-rabbit antibody (ab97051; 1:2000, Abcam), protein bands were detected by chemiluminescence using the ECL Prime western blotting detection reagent (GE Healthcare, Little Chalfont, UK). Images were acquired on a charge-coupled device camera system (ImageQuant LAS 4000; GE Healthcare).

The ratio of phosphorylated to total proteins was quantitated using ImageJ software (NIH, Frederick, MD, USA). The membranes used for immunoblotting against FOXO1, NF-κB, MuRF1, PGC1α, CD31, and IRS-2 were reused after treatment with stripping buffer (ATTO WSE-7240 EzReprobe, ATTO Cop., Tokyo, Japan). Specifically, they were incubated with the anti-α-tubulin antibody, and protein expression was normalized to that of α-tubulin. Entire images of western blotting are shown in online Additional file 1: Fig. S2-9.

### Evaluation of oxidative stress in muscle

Protein carbonyls were used to evaluate oxidative stress and were quantified in an OxiSelect Protein Carbonyl ELISA Kit (Cell Biolabs, Inc., San Diego, CA, USA) using 10 μg of extracted protein [20, 22].

### Statistical analysis

All values are expressed as means ± standard error of the mean (SE). *P* < 0.05 was considered statistically significant. Following the Kruskal–Wallis test, differences among the four groups were compared using the Mann–Whitney *U* test. All statistical analyses were performed using JMP software version 13.0.0 (SAS Institute, Cary, NC, USA).

## Results

### Exercise did not upregulate protein expressions of IGF-1 and Akt phosphorylation in EDL and soleus muscles of SDT fatty rats.

**Fig. 1:**
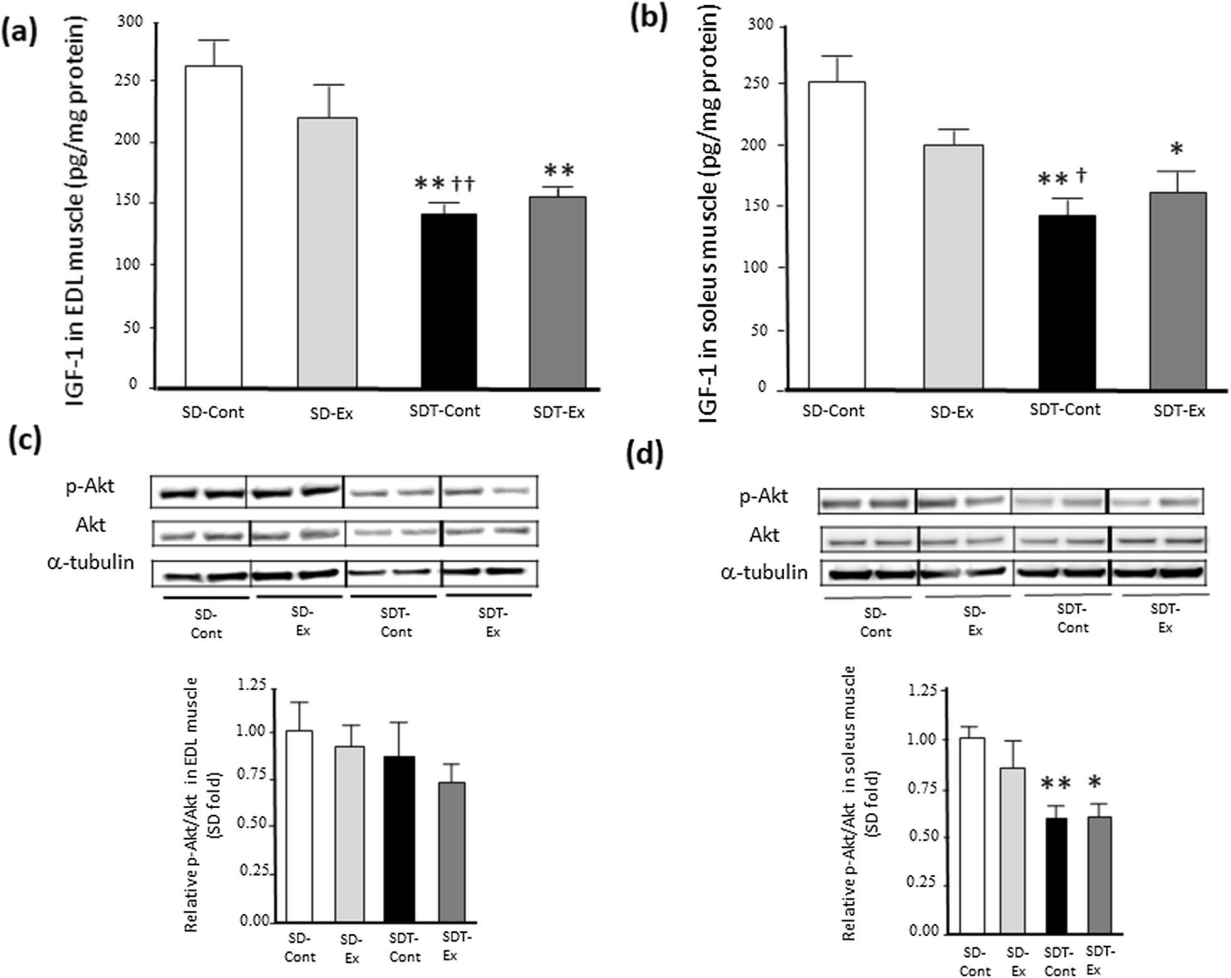
Protein expression of insulin-like growth factor-1 (IGF-1) and phosphorylated Akt related to muscle hypertrophy in the extensor digitorum longus (EDL) and soleus muscles. Protein expression of IGF-1 (**a**: EDL, **b**: soleus) and phosphorylated Akt (**c**: EDL, **d**: soleus) in the EDL and soleus muscles as determined by western blot analysis. Entire images of western blotting are shown in online Additional file 1: Fig S2. Values are presented as mean ± standard error of the mean. SD-Cont group, n = 5; SD-Ex group, n = 5; SDT-Cont group, n = 6; and SDT-Ex group, n = 6. ^*^P < 0.05 and ^**^P < 0.01 vs. the SD-Cont group; ^†^*P* < 0.05 and ^††^*P* < 0.01 vs. the SD-Ex group

Given that muscle IGF-1 and Akt phosphorylation have been reported to play an important role in muscle hypertrophy [23], we evaluated protein expression of IGF-1 and Akt phosphorylation. The IGF-1 protein expression in the EDL (Fig. 1a) and soleus (Fig. 1b) muscles of the SDT control (SDT-Cont) group was significantly lower than that of the SD control (SD-Cont) (p < 0.01) and SD exercise (SD-Ex) (EDL, p < 0.01; soleus, p < 0.05) groups. The IGF-1 protein expression in the EDL (Fig. 1a) and soleus (Fig. 1b) muscles of the SDT exercise (SDT-Ex) group was significantly lower than that of the SD-Cont group (EDL, p < 0.01; soleus, p < 0.05). There were no statistically significant differences in IGF-1 protein expression between the SDT-Cont and SDT-Ex groups in each EDL (Fig. 1a) and soleus (Fig. 1b) muscle.

Whereas there were no significant differences in the protein expression of Akt phosphorylation in the EDL muscle among all rats (Fig. 1c), the Akt phosphorylation in the soleus muscle of the SDT-Cont (p < 0.01) and SDT-Ex (p < 0.05) groups was significantly lower than that of the SD-Cont group (Fig. 1d). There were no statistically significant differences in the Akt phosphorylation between the SDT-Cont and SDT-Ex groups in each EDL (Fig. 1c) and soleus (Fig. 1d) muscle.

### Exercise did not affect the protein expression of S6K phosphorylation related to activation of muscle protein synthesis in EDL and soleus muscles of SDT fatty rats.

**Fig. 2:**
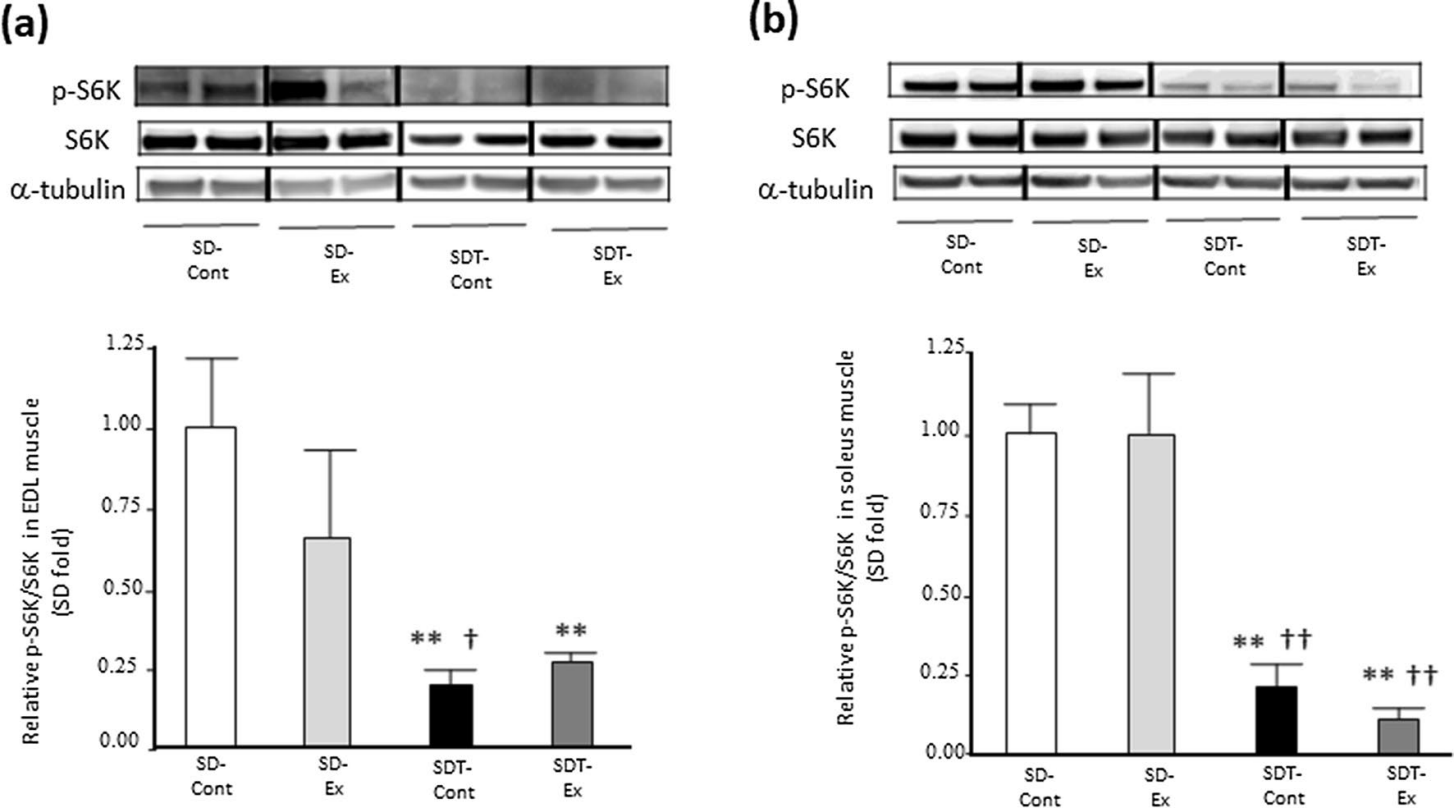
Protein expression of phosphorylated ribosomal protein S6 kinase beta-1 (S6K) related to protein synthesis in the extensor digitorum longus (EDL) and soleus muscles. Protein expression of phosphorylated ribosomal protein S6 kinase beta-1 (S6K) (**a**: EDL, **b**: soleus) in the EDL and soleus muscles as determined by western blot analysis. Entire images of western blotting are shown in online Additional file 1: Fig S3. Values are presented as mean ± standard error of the mean. SD-Cont group, n = 5; SD-Ex group, n = 5; SDT-Cont group, n = 6; and SDT-Ex group, n = 6. ^**^P < 0.01 vs. the SD-Cont group; ^†^*P* < 0.05 and ^††^*P* < 0.01 vs. the SD-Ex group

Regarding a signaling pathway of muscle protein synthesis, we evaluated S6K activation. The phosphorylation levels of S6K in the EDL (Fig. 2a) and soleus (Fig. 2b) muscles of the SDT-Cont group were significantly lower than those of the SD-Cont (p < 0.01) and SD-Ex (EDL, p < 0.05; soleus, p < 0.01) groups. Whereas the level in the EDL muscle of the SDT-Ex group was significantly lower than that of the SD-Cont group (p < 0.01) (Fig. 2a), the level in the soleus muscle of the SDT-Ex group was significantly lower than those of the SD-Cont and SD-Ex groups (p < 0.01) (Fig. 2b). There were no statistically significant differences in the S6K phosphorylation between the SDT-Cont and SDT-Ex groups in each EDL (Fig. 2a) and soleus (Fig. 2b) muscle.

### Exercise declined the expression of FOXO1 in EDL muscle of SDT fatty rats.

**Fig. 3:**
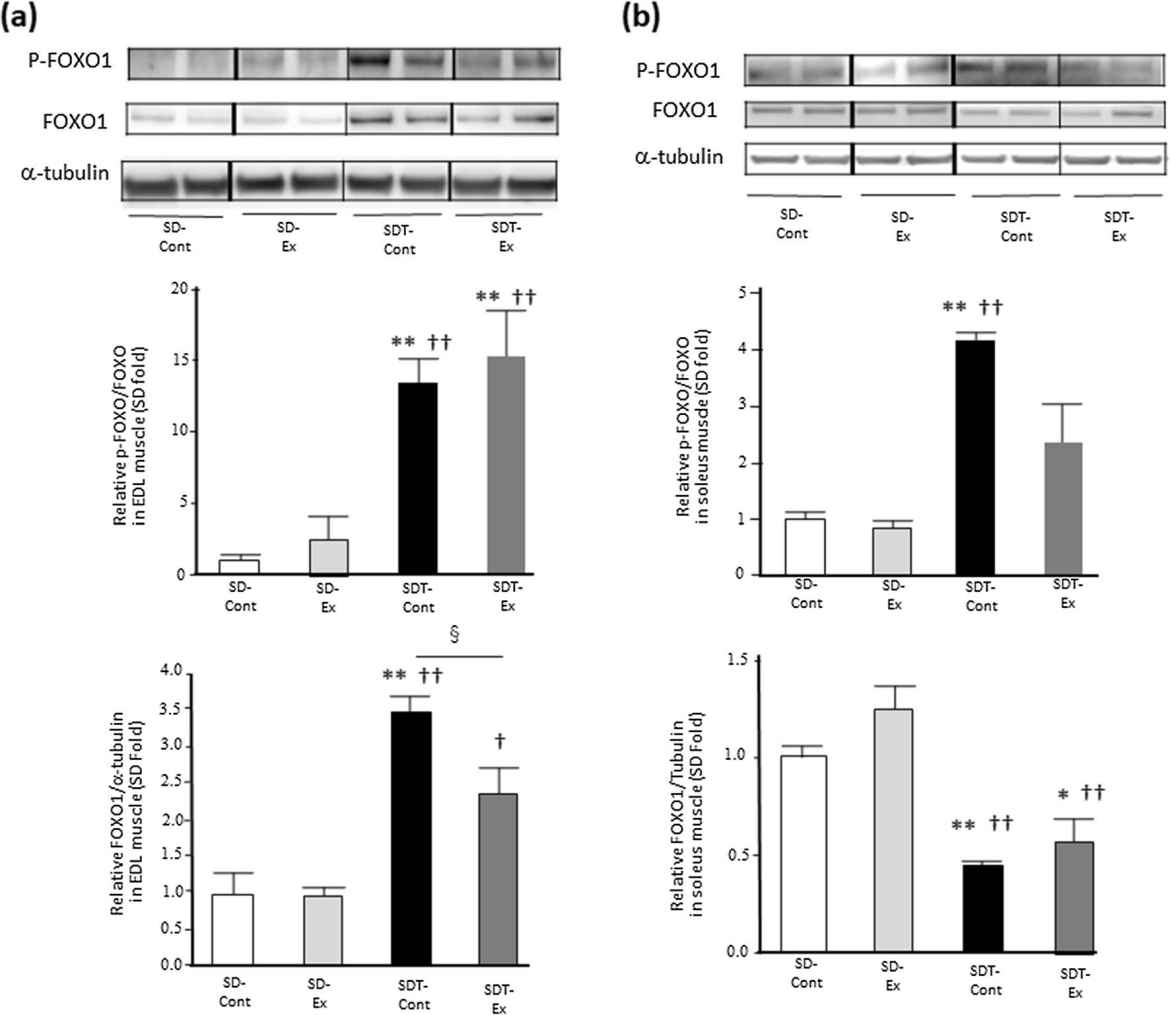
Protein expression of phosphorylated forkhead box protein O1 (FOXO1) related to protein degradation in the extensor digitorum longus (EDL) and soleus muscles. Protein expression of phosphorylated forkhead box protein O1 (FOXO1) (**a**: EDL, **b**: soleus) in the EDL and soleus muscles as determined by western blot analysis. Entire images of western blotting are shown in online Additional file 1: Fig. S4. Values are presented as mean ± standard error of the mean. SD-Cont group, n = 5; SD-Ex group, n = 5; SDT-Cont group, n = 6; and SDT-Ex group, n = 6. ^*^P < 0.05 and ^**^P < 0.01 vs. the SD-Cont group, ^†^*P* < 0.05 and ^††^*P* < 0.01 vs. the SD-Ex group, and ^§^*P* < 0.05 and ^§§^*P* < 0.01 vs. the SDT-Cont group

To evaluate a signaling pathway of muscle protein degradation, the expression of both FOXO1 and phosphorylated FOXO1 was evaluated. The phosphorylation levels of FOXO1 in EDL muscles of both SDT-Cont and SDT-Ex groups were significantly higher than those of the SD-Cont (p < 0.01) and SD-Ex (p < 0.01) groups (Fig. 3a). There were no statistically significant differences in the FOXO1 phosphorylation between the SDT-Cont and SDT-Ex groups in EDL muscle (Fig. 3a). The protein expression of FOXO1 in the EDL muscle of the SDT-Cont group was significantly higher than that in all the SD rats (p < 0.01) (Fig. 3a), and the level in the SDT-Ex group was significantly higher than that in the SD-Ex group (p < 0.05) (Fig. 3a). The protein expression in the EDL muscle of the SDT-Ex group was significantly lower than that in the SDT-Cont group (p < 0.05) (Fig. 3a).

The phosphorylation level of FOXO1 in soleus muscle of SDT-Cont group was significantly higher than those of the SD-Cont (p < 0.01) and SD-Ex (p < 0.01) groups (Fig. 3b). There were no statistically significant differences in the FOXO1 phosphorylation between the SDT-Cont and SDT-Ex groups in the soleus muscle (Fig. 3b). The protein expression of FOXO1 in the soleus muscle of the SDT-Cont (p < 0.01) and SDT-Ex (p < 0.05 vs SD-Cont, p < 0.01 vs SD-Ex) groups was significantly lower than that in all SD rats (Fig. 3b). There was no statistically significant difference in the FOXO1 protein expression between the SDT-Cont and SDT-Ex groups in the soleus muscle (Fig. 3b).

### ***Protein expression of NF-***κ***B phosphorylation was upregulated in EDL muscle of SDT fatty rats.***

**Fig. 4:**
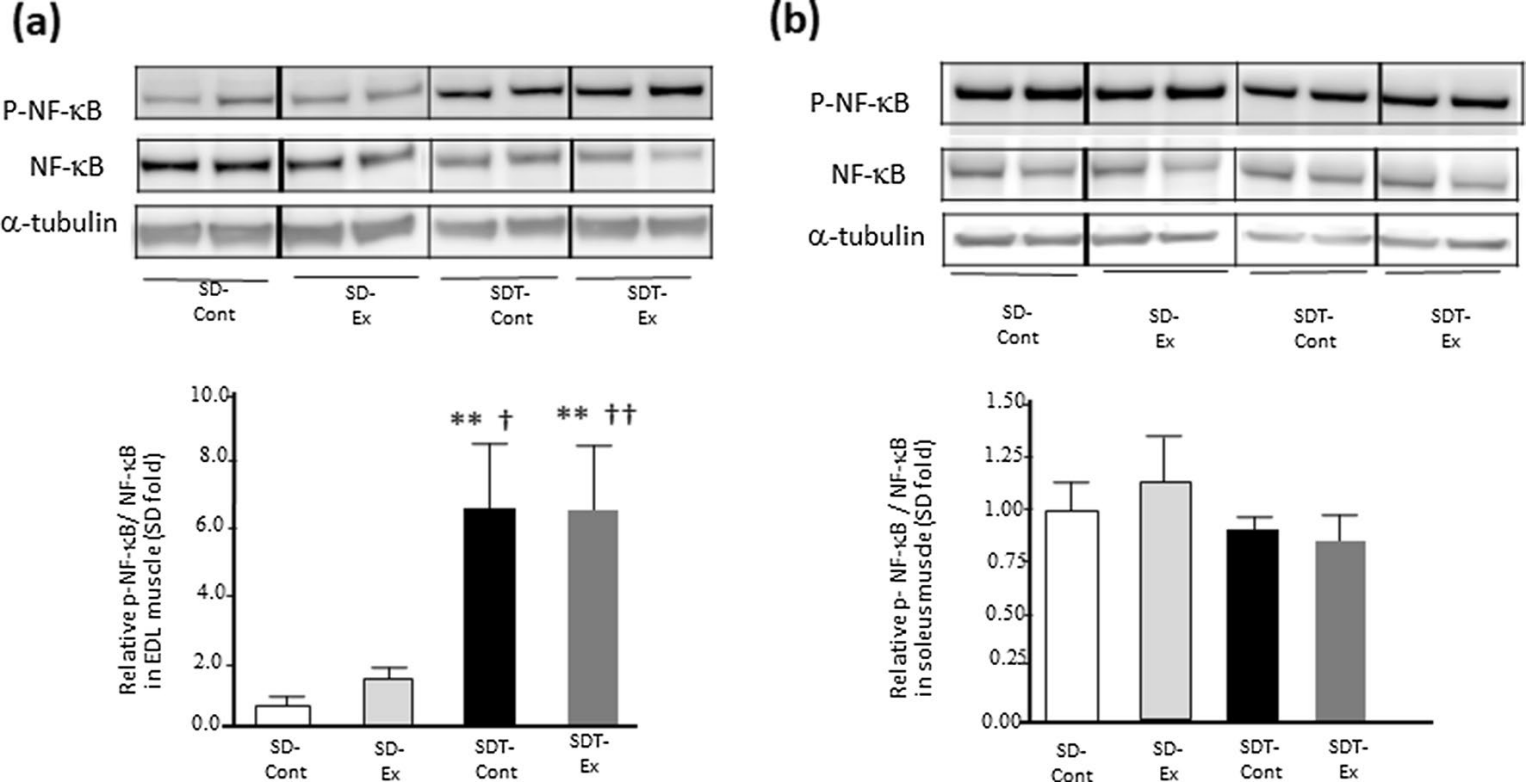
Protein expression of phosphorylated Nuclear Factor-κB (NF-κB) in the extensor digitorum longus (EDL) and soleus muscles. Protein expression of phosphorylated Nuclear Factor-κB (NF-κB) (**a**: EDL, **b**: soleus) in the EDL and soleus muscles as determined by western blot analysis. Entire images of western blotting are shown in online Additional file 1: Fig. S5. Values are presented as mean ± standard error of the mean. SD-Cont group, n = 5; SD-Ex group, n = 5; SDT-Cont group, n = 6; and SDT-Ex group, n = 6. ^*^P < 0.05 and ^**^P < 0.01 vs. the SD-Cont group, ^†^*P* < 0.05 and ^††^*P* < 0.01 vs. the SD-Ex group, and ^§^*P* < 0.05 and ^§§^*P* < 0.01 vs. the SDT-Cont group

As the activation of NF-κB is related to upregulation of MuRF1 expression [24], the level of NF-κB phosphorylation was evaluated. The phosphorylation levels of NF-κB in EDL muscles of both SDT-Cont and SDT-Ex groups were significantly higher than those of the SD-Cont (p < 0.01) and SD-Ex (p < 0.01) groups (Fig. 4a). There were no statistically significant differences in the NF-κB phosphorylation between the SDT-Cont and SDT-Ex groups in EDL muscle (Fig. 4a). In soleus muscles, there were no significant differences in the phosphorylation levels among all rats (Fig. 4b).

### Exercise decreased both gene and protein expressions of MuRF1 in EDL muscle of SDT fatty rats.

**Fig. 5:**
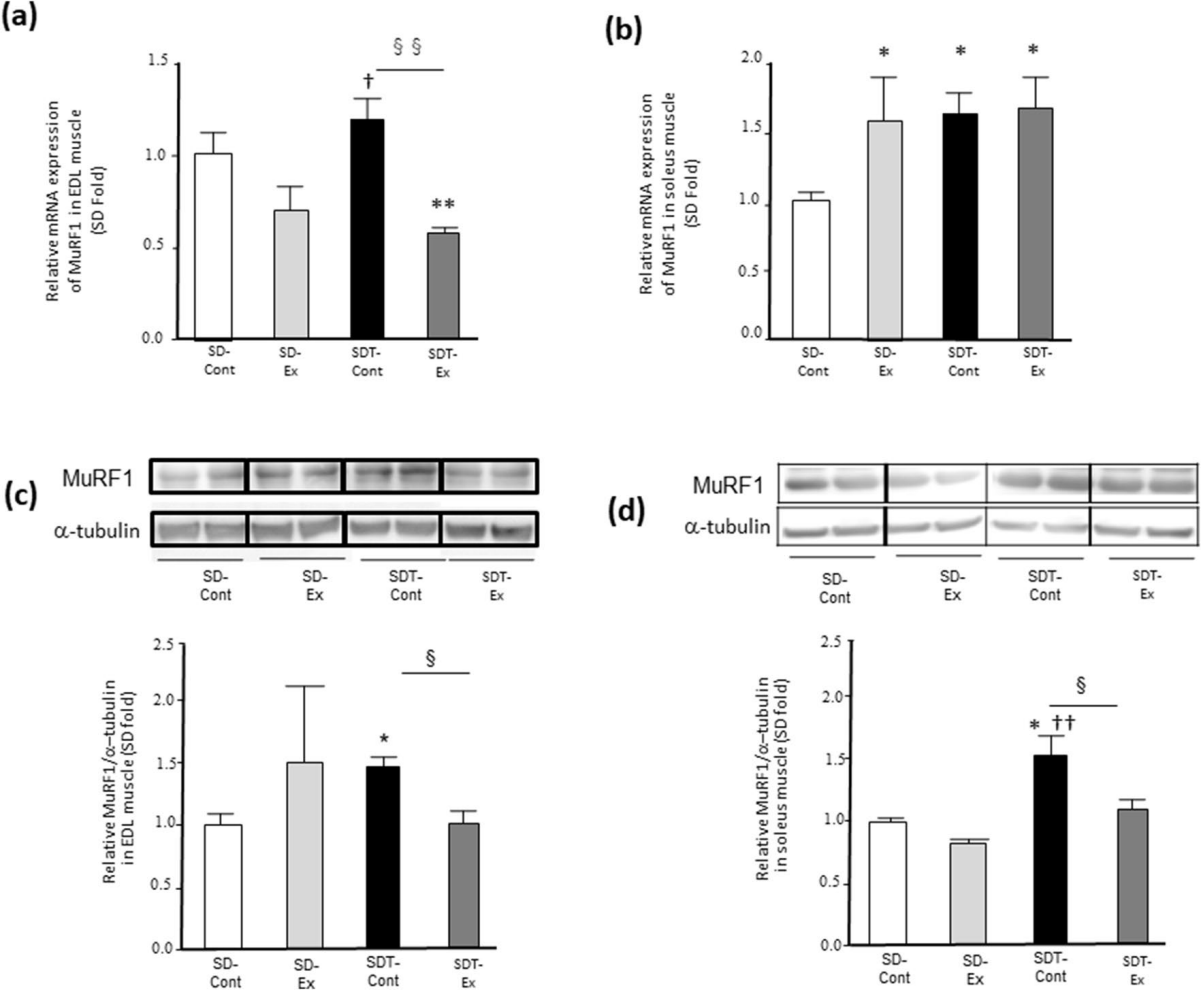
Gene and protein expressions of muscle RING-finger protein-1 (MuRF1) in the extensor digitorum longus (EDL) and soleus muscles. Gene expression of *MuRF1* as determined by real-time quantitative reverse transcription polymerase chain reaction (**a**: EDL, **b**: soleus) and protein expression of MuRF1 by western blot analysis (**c**: EDL, **d**: soleus). Entire images of western blotting are shown in online Additional file 1: Fig S6. Values are presented as mean ± standard error of the mean. SD-Cont group, n = 5; SD-Ex group, n = 5; SDT-Cont group, n = 6; and SDT-Ex group, n = 6. ^*^P < 0.05 and ^**^P < 0.01 vs. the SD-Cont group, ^†^*P* < 0.05 and ^††^*P* < 0.01 vs. the SD-Ex group, and ^§^*P* < 0.05 and ^§§^*P* < 0.01 vs. the SDT-Cont group

Although the MuRF1 gene expression in the EDL muscles of the SDT-Cont group was significantly higher than that of the SD-Ex group (p < 0.05) (Fig. 5a), the expression in the SDT-Ex group was significantly lower than that in the SD-Cont group (p < 0.01) (Fig. 5a). The MuRF1 gene expression in the SDT-Ex group was significantly lower than that in the SDT-Cont group (p < 0.01) (Fig. 5a). The MuRF1 gene expression in the soleus muscles of the SD-Ex, SDT-Cont, and SDT-Ex groups was significantly higher than that in the SD-Cont group (p < 0.05) (Fig. 5b).

The MuRF1 protein expression in the EDL muscle of the SDT-Cont group was significantly higher than that of the SD-Cont group (p < 0.05) (Fig. 5c), and the expression in the SDT-Ex group was significantly lower than that in the SDT- Cont group (p < 0.05) (Fig. 5c). In the soleus muscles, the MuRF1 protein expression in the SDT-Cont group was significantly higher than that of the SD-Cont (p < 0.05) and SD-Ex groups (p < 0.01) (Fig. 5d), and the expression in the SDT-Ex group was significantly lower than that in the SDT-Cont group (p < 0.05) (Fig. 5d).

### ***Exercise did not change the protein expression of PGC-1***α ***whose molecules is related to mitochondrial biosynthesis, in EDL and soleus muscles of SDT fatty rats***

**Fig. 6:**
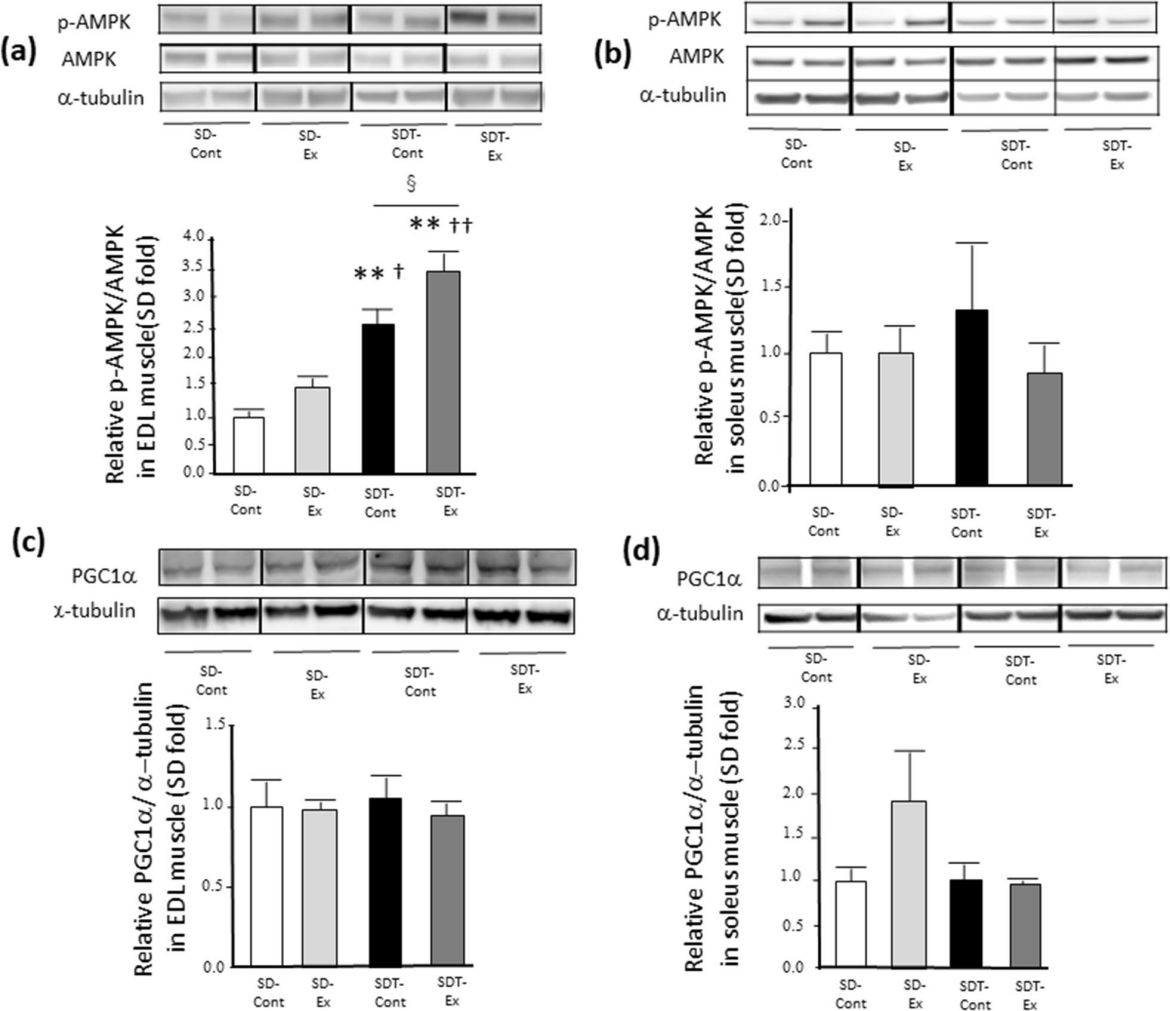
Protein expression of phosphorylated AMP-activated protein kinase (AMPK) and proliferator-activated receptor gamma-coactivator-1 alpha (PGC-1α) in the extensor digitorum longus (EDL) and soleus muscles. Phosphorylated AMPK (a: EDL, b: soleus) and PGC-1α protein expression (c: EDL, d: soleus) in the EDL and soleus muscles as determined by western blot analysis. Entire images of western blotting are shown in online Additional file 1: Fig. S7 and S8. Values are presented as mean ± standard error of the mean. SD-Cont group, n = 5; SD-Ex group, n = 5; SDT-Cont group, n = 6; and SDT-Ex group, n = 6. ^**^P < 0.01 vs. the SD-Cont group, ^†^*P* < 0.05 and ^††^*P* < 0.01 vs. the SD-Ex group, and ^§^*P* < 0.05 vs. the SDT-Cont group

Exercise-accelerated AMPK phosphorylation [25] has been related to mitochondrial biosynthesis [26]. We therefore evaluated AMPK phosphorylation (Fig. 6a, b), the expression of protein related to mitochondrial biosynthesis, the protein expression of PGC-1α(Fig. 6c, d).

The AMPK phosphorylation levels in the EDL muscle of all SDT fatty rats were significantly increased compared with those in all SD rats (SDT-Cont, p < 0.01 vs SD-Cont, p < 0.05 vs SD-Ex; SDT-Ex, p < 0.01 vs all SD rats) (Fig. 6a), and the levels in the SDT-Ex group were significantly increased compared with those in the SDT-Cont group (p < 0.05) (Fig. 6a). In the soleus muscle, there were not significantly differences in the AMPK phosphorylation levels in all rats (Fig. 6b).

Regarding PGC-1αprotein expression, there was no statistically significant difference in each EDL (Fig. 6c) and soleus (Fig. 6d) muscle in all rats.

### Exercise increased the protein expression of CD31, IRS2, and eNOS phosphorylation related to angiogenesis in EDL muscle of SDT fatty rats, but not soleus muscle.

**Fig. 7:**
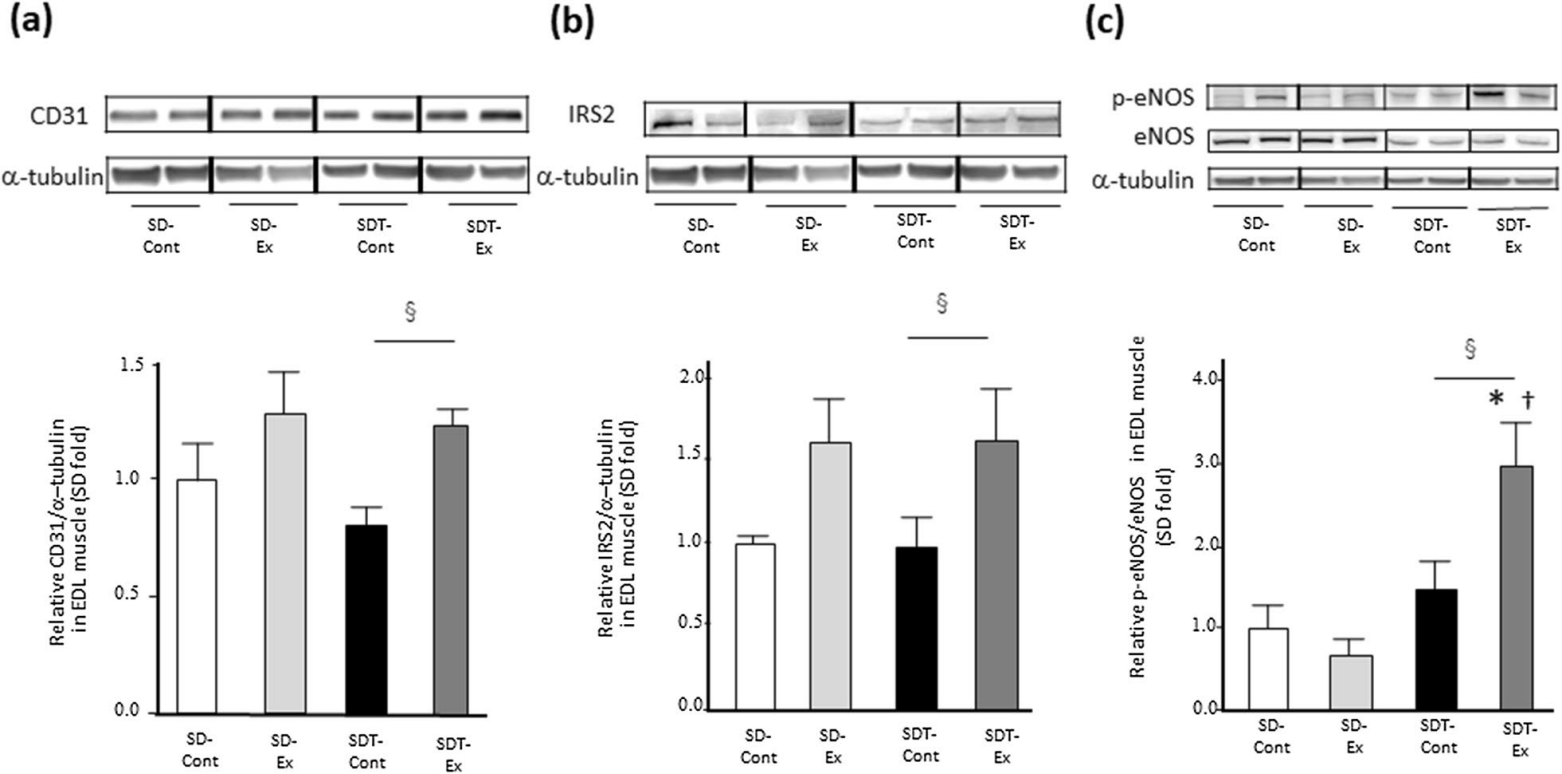
Protein expression related to vascular endothelial cells in the extensor digitorum longus (EDL) muscle. Protein expression of CD31 (**a**), insulin receptor substrate 2 (IRS2) (**b**), and phosphorylated endothelial nitric oxide synthase (eNOS) (c) as determined by western blot analysis. Entire images of western blotting are shown in online Additional file 1: Fig. S9 (a, c, e) and S10 (a, c, e). Values are presented as mean ± standard error of the mean. SD-Cont group, n = 5; SD-Ex group, n = 5; SDT-Cont group, n = 6; and SDT-Ex group, n = 6. ^*^P < 0.05 vs. the SD-Cont group, ^†^*P* < 0.05 vs. the SD-Ex group, and ^§^*P* < 0.05 vs. the SDT-Cont group

**Fig. 8:**
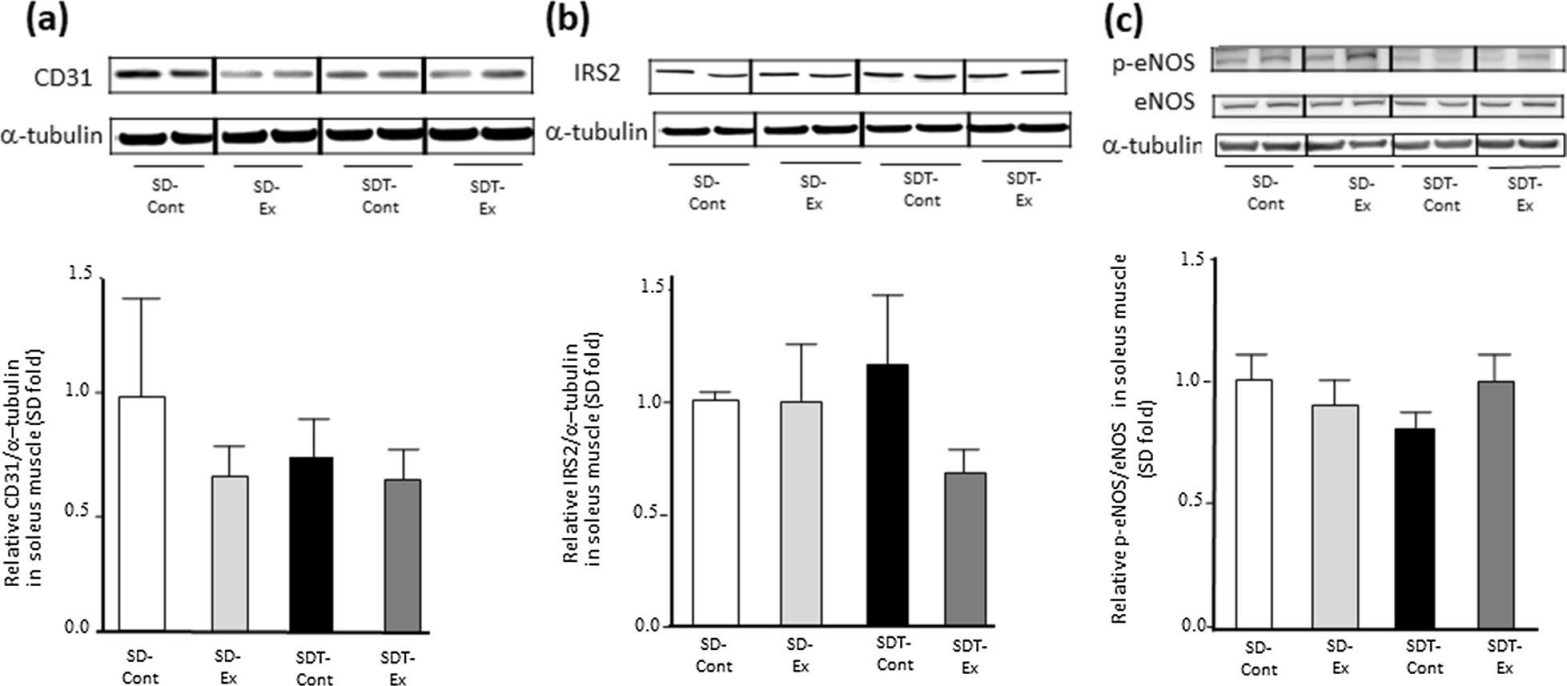
Protein expression related to vascular endothelial cells in the soleus muscle. Protein expression of CD31 (**a**), insulin receptor substrate 2 (IRS2) (**b**), and phosphorylated endothelial nitric oxide synthase (eNOS) (c) as determined by western blot analysis. Entire images of western blotting are shown in online Additional file 1: Figure S9 (b, d, f) and S10 (b, d, f). Values are presented as mean ± standard error of the mean

Given that exercise was reported to increase vascularity in muscles [27], we evaluated the protein expression of CD31 and IRS2 expressed in vascular endothelial cells and the eNOS phosphorylation related to the activation of endothelial cells in EDL and soleus muscles. In the EDL muscle, the protein expression of CD31 (p < 0.05) (Fig. 7a) and IRS2 (p < 0.05) (Fig. 7b) was significantly higher in the SDT-Ex group than in the SDT-Cont group. eNOS phosphorylation in the SDT-Ex group was significantly higher than that in the SD-Cont, SD-Ex, and SDT-Cont groups (p < 0.05) (Fig. 7c). In the soleus muscle, there were no statistically significant differences in their gene expression levels among all rats (Fig. 8a, b, c).

### Oxidative stress evaluated by carbonylated protein was not increased in EDL and soleus muscles of SDT fatty rats.

**Fig. 9:**
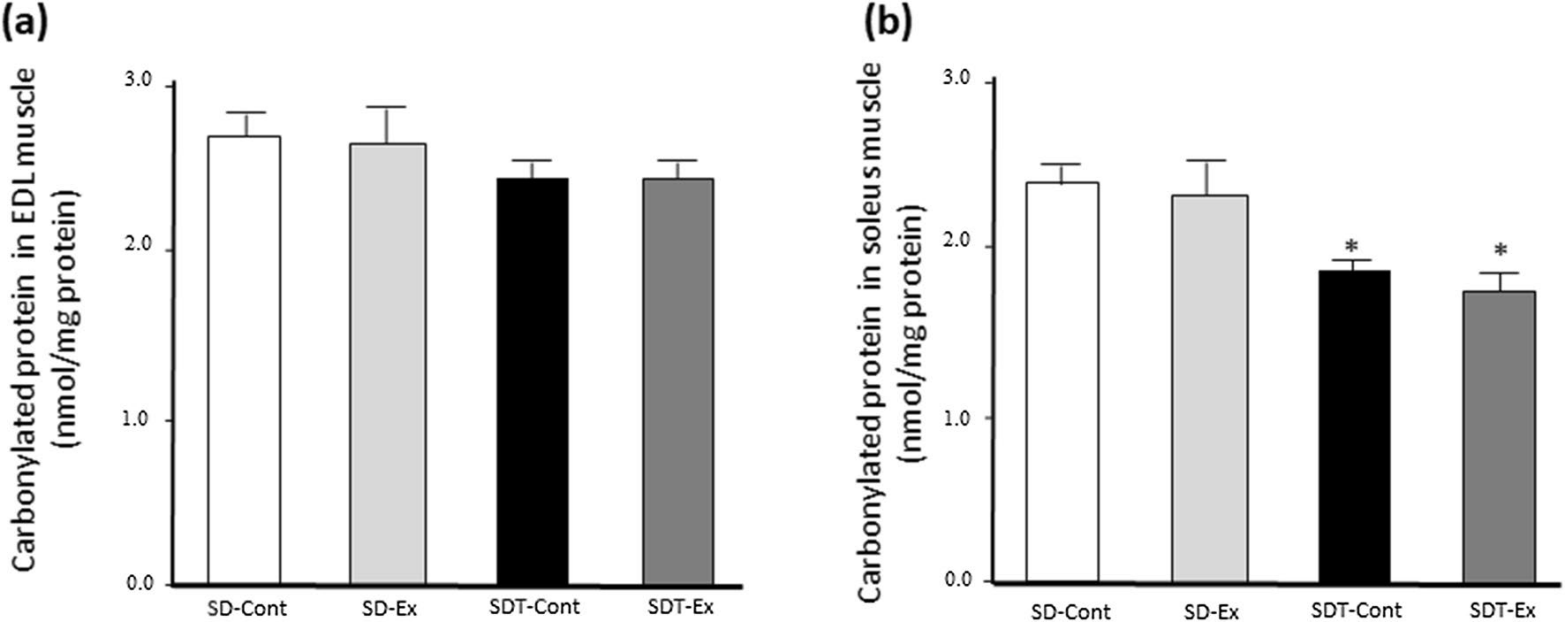
Carbonylated protein in the extensor digitorum longus (EDL) and soleus muscles. Carbonylated protein in EDL (**a**) and soleus (**b**) muscles. Values are presented as mean ± standard error of the mean. SD-Cont group, n = 5; SD-Ex group, n = 5; SDT-Cont group, n = 6; and SDT-Ex group, n = 6. ^*^P < 0.05 vs. the SD-Cont group

Given that exercise has been related to the alleviation of oxidative stress, we evaluated carbonylated protein as a marker of oxidative stress. Although there were no significant differences in protein carbonyl levels in the EDL among all rats (Fig. 9a), its levels in the soleus muscle of all SDT fatty rats were significantly lower than that of SD-Cont group (p < 0.05) (Fig. 9b).

## Discussion

**Fig. 10:**
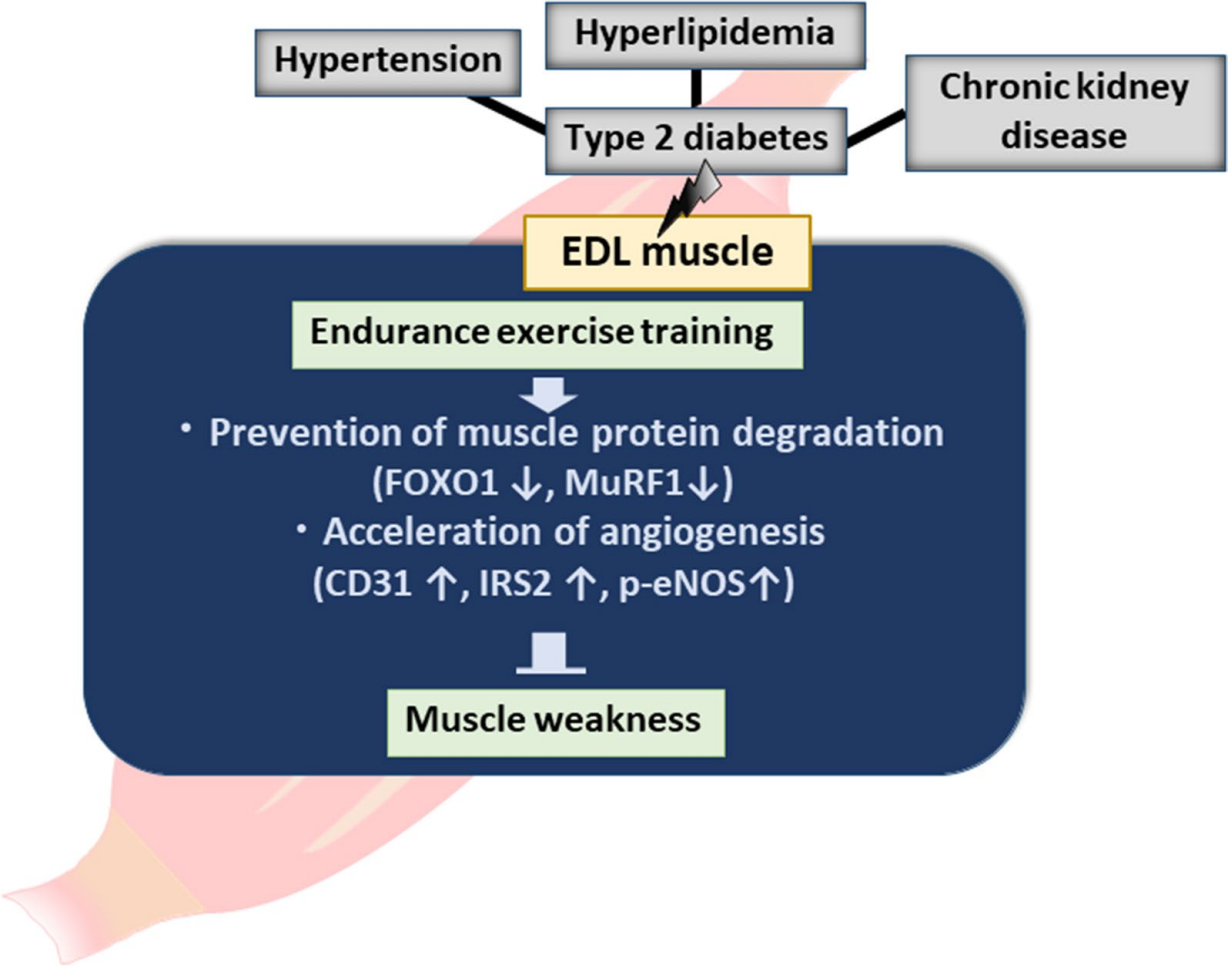
Effective mechanisms of endurance exercise training on the extensor digitorum longus (EDL) muscle in rats with type 2 diabetes complicated by obesity, hypertension, and hyperlipidemia. The previous study indicated that endurance exercise training enhanced the muscle strength and cross-sectional area of the type IIb muscle fibers of the EDL in type 2 diabetes with hypertension, hyperlipidemia, and chronic kidney disease (Additional file 1: Table S1–3, Fig. S1) [18]. As a mechanism, the present study revealed that the exercise training could improve muscle weakness by preventing muscle degradation and accelerating angiogenesis in the EDL

Our previous study showed that the exercise training increased the muscle strength and cross-sectional area of the type IIb muscle fibers in the EDL and ameliorated hypertension, hyperlipidemia, and CKD in SDT fatty rats although the endurance exercise training did not significantly change blood glucose levels and the cross-sectional area of the type I muscle fibers in the soleus muscle (Additional file 1: Table S1–3 and Additional file 1: Fig. S1) [18]. As a mechanism for preventing muscle wasting due to the exercise training in the EDL muscle SDT fatty rats, the present study revealed that the exercise training downregulated the expression of FOXO1 and MuRF1 and increased the expression of CD31, IRS2, and phosphorylated eNOS in the EDL muscle of the SDT fatty rats. The exercise training did not change the protein expression of PGC-1α despite the increased expression of phosphorylated AMPK in the SDT fatty rats. Thus, the exercise training helps in preventing muscle protein degradation and in accelerating the angiogenic response in the EDL, regardless of blood glucose levels in type 2 diabetes with hypertension, hyperlipidemia, and CKD (Fig. 10).

Muscle weakness is caused by a disrupted balance between protein synthesis and protein degradation. In diabetes, muscle IGF-1 expression plays an important role in muscle protein synthesis [28]. Reduced IGF-1 expression results in muscle atrophy [29], which was reportedly prevented by exercise [9]. In the present study, markedly suppressed expression of both muscle IGF-1 and phosphorylated S6K, but not downregulated Akt phosphorylation, were observed in the EDL of the SDT fatty rats and were not restored by exercise training. Given that the present exercise training did not significantly decrease blood glucose levels in the SDT fatty rats [18], we considered that muscle IGF-1 expression was not increased by the exercise training because the intensity of the exercise training might have been mild. In addition, AMPK phosphorylation was upregulated in the EDL of the SDT fatty rats, with and without the exercise, compared with that in the SD rats, and the exercise increased AMPK phosphorylation in the SDT fatty rats. AMPK is activated in response to a decrease in cellular adenosine triphosphate (ATP), leading to the development of catabolic processes by the suppression of protein synthesis for generating ATP [30, 31]. Thus, the present exercise might not activate protein synthesis because the exercise did not induce IGF-1 upregulation and increased AMPK phosphorylation in the SDT fatty rats.

By contrast, the present study showed that exercise training downregulated the expression of FOXO1 and MuRF1 related to protein degradation in the EDL of the SDT fatty rats although the exercise training did not significantly change the levels of FOXO1 phosphorylation induced in the EDL of the SDT fatty rats. In addition, NF-κB phosphorylation, which leads to the upregulation of MuRF1 expression [24], was elevated in the EDL of the SDT fatty rats, but the exercise did not suppress the NF-κB phosphorylation. FOXO1 plays a central role in MuRF1-mediated proteolysis [32]. FOXO1 expression in skeletal muscle has been reported to be significantly upregulated in patients with type 2 diabetes compared with healthy individuals [33] and to be decreased by resistance-training exercises in the skeletal muscle of healthy individuals [34]. Hyperlipidemia [35] and CKD [36], which were observed in the SDT fatty rats and were attenuated by the exercise training [18], were reported to increase muscle FOXO1 expression. Type 2 diabetes, hyperlipidemia, and CKD can contribute to FOXO1 upregulation in the EDL of SDT fatty rats. Regarding MuRF1 expression, FOXO1 primarily upregulates MuRF1 expression in type II fibers because MuRF1 is enriched in type II fibers [37, 38], a finding supported by the present study. Moreover, previous evidence has shown that exercise prevents muscle protein degradation via AMPK-PGC1α signaling related to mitochondrial biosynthesis [10] or oxidative stress [11]. However, upregulated expression of PGC1α, whose molecules are related to mitochondrial biosynthesis, were not observed in the SDT fatty rats. These results indicated that the exercise training was not associated with the increase in mitochondria content in the EDL of the SDT fatty rats. Oxidative stress was also not involved in muscular atrophy because no change was observed in carbonylated protein levels in the EDL of the SDT fatty rats. Thus, the present exercise training might prevent muscle atrophy by downregulating the FOXO1–MuRF1 pathway in the EDL of the SDT fatty rats.

Exercise training has been reported to accelerate the angiogenic response in skeletal muscle [39], a finding supported by the present study. As a mechanism between exercise training and angiogenesis, it has been reported that exercise training enhances the angiogenic responses via the release of exosomes with beneficial effects on endothelial cells into the circulation from the skeletal muscle in type 2 diabetic mice [13]. Moreover, hypertension in the SDT fatty rats was ameliorated by exercise training [18]. Given that hypertension affects vascular endothelial function [40], the exercise training might provide favorable antihypertensive effects on the endothelial cells. Further study is needed to clarify the relationship between acceleration of angiogenesis and prevention of muscle weakness due to exercise training in the SDT fatty rats.

Our previous study observed no increase in muscle volume or cross-sectional area of the type I muscle fibers in the soleus muscle of the SDT fatty rats from the treadmill exercise [18] although our present study showed that the exercise reduced upregulated MuRF1 protein expression in the soleus muscle of the SDT fatty rats. The exercise did not alleviate suppressed expression of IGF-1, phosphorylated Akt, and S6K protein, and did not activate mitochondrial biosynthesis in the soleus muscle of the SDT fatty rats. In addition, the exercise did not induce increase in angiogenic response. Furthermore, decreased oxidative stress was observed in the soleus muscle of the SDT fatty rats. As antioxidative activity was reported to reduce the beneficial effects of exercise on muscle [12], some oxidative stress may be needed for type I muscle fibers in the soleus muscle of the SDT fatty rats. Kondo et al. also reported that low-intensity treadmill exercise (15 m/min, 60 min/session, 5 sessions/week for 3 weeks) did not increase the muscle volume of the soleus muscle in Goto-Kakizaki rats, another type 2 diabetic model [41]. On the contrary, voluntary running exercise was reported to increase the cross-sectional area of the type I muscle fibers of the soleus muscle but not of the type IIb muscle fibers of the plantaris muscle in diabetic Goto-Kakizaki rats [42]. Each treadmill exercise and voluntary running exercise might influence the separate muscle fibers in type 2 diabetes, and mild treadmill exercise might not contribute to the enlargement of type I muscle fibers. Although the treadmill exercise did not change the blood glucose levels (Additional file 1: Figure S1)[18, 41], the voluntary running exercise did reduce the glucose levels [42]. Given that hyperglycemia has been reported to affect type I muscle fibers more severely than type IIb muscle fibers by reducing the glucose transporter system in type 2 diabetes [43], the treadmill exercise intensity that attenuates hyperglycemia might need to be determined to provide the most favorable effect on type I muscle fibers.

## Conclusion

Endurance exercise training might alleviate muscle wasting by preventing muscle degradation through the inhibition of the FOXO1–MuRF1 pathway and by increasing the angiogenic response in the EDL muscle in type 2 diabetes with hypertension, hyperlipidemia, and CKD.

### Supplementary Information

**Additional file 1: Table S1** Muscle strength at 8-, 12-, and 16-week-old. **Table S2 **Muscle weight in 16-week-old SD and SDT fatty rats. **Table S3 **Cross-sectional area of type I and type IIb muscle fibers in 16-week-old SD and SDT fatty rats. **Figure S1**. Changes in body weight (a) and blood glucose levels (b) at 8-, 12-, and 16-week-old. SD-Cont group, n = 5; SD-Ex group, n = 5; SDT-Cont group, n = 6; and SDT-Ex group, n = 6. Values are presented as the mean ± standard error of the mean (SEM). *P < 0.05 and **P < 0.01 versus the SD group at the same age; # *P *< 0.05 and ## *P *< 0.01 versus the SD-Ex group at the same age; †*P *< 0.05 and ††*P *< 0.01 versus the same group at 8-week-old. These figures were created based on the results of reference No.18. **Figure S2 **Western blot analysis of p-Akt in EDL (a) and soleus (b) muscles, Akt in EDL (c) and soleus (d) muscles, and α-tubulin in EDL (e) and soleus (f) muscles on the same membrane in each group. Red lines represent the edge of each cut membrane. Red boxes show the regions of the original blots used in main figures. **Figure S3** Western blot analysis of p-S6K in EDL (a) and soleus (b) muscles, S6K in EDL (c) and soleus (d) muscles, and α-tubulin in EDL (e) and soleus (f) muscles on the same membrane in each group. Red lines represent the edge of each cut membrane. Red boxes show the regions of the original blots used in main figures. **Figure S4** Western blot analysis of p-Foxo1 in EDL (a) and soleus (b) muscles, Foxo1 in EDL (c) and soleus (d) muscles and α-tubulin in EDL (e) and soleus (f) muscles on the same membrane in each group. Red lines represent the edge of each cut membrane. Red boxes show the regions of the original blots used in main figures. **Figure S5** Western blot analysis of p-NF-B in EDL (a) and soleus (b) muscles, NF-B in EDL (c) and soleus (d) muscles and α-tubulin in EDL (e) and soleus (f) muscles on the same membrane in each group. Red lines represent the edge of each cut membrane. Red boxes show the regions of the original blots used in main figures. **Figure S6** Western blot analysis of MuRF1 in EDL (a) and soleus (b) muscles, and α-tubulin in EDL (c) and soleus (d) muscles on the same membrane in each group. Red lines represent the edge of each cut membrane. Red boxes show the regions of the original blots used in main figures. **Figure S7** Western blot analysis of p-AMPK in EDL (a) and soleus (b) muscles, AMPK in EDL (c) and soleus (d) muscles, and α-tubulin in EDL (e) and soleus (f) muscles on the same membrane in each group. Red lines represent the edge of each cut membrane. Red boxes show the regions of the original blots used in main figures. **Figure S8** Western blot analysis of PGC1 in EDL (a) and soleus (b) muscles, and α-tubulin in EDL (c) and soleus (d) muscles on the same membrane in each group. The red lines represent the edge of each cut membrane. Red lines represent the edge of each cut membrane. Red boxes show the regions of the original blots used in main figures. **Figure S9** Western blot analysis of CD31 in EDL (a) and soleus (b) muscles, IRS2 in EDL (c) and soleus (d) muscles, and α-tubulin in EDL (e) and soleus (f) muscles on the same membrane in each group. Red lines represent the edge of each cut membrane. Red boxes show the regions of the original blots used in main figures. **Figure S10** Western blot analysis of p-eNOS in EDL (a) and soleus (b) muscles, eNOS in EDL (c) and soleus (d) muscles, and α-tubulin in EDL (e) and soleus (f) muscles on the same membrane in each group. Red lines represent the edge of each cut membrane. Red boxes show the regions of the original blots used in main figures.

## Data Availability

The datasets supporting the conclusions of this article are available in the repository of the corresponding author (AK-I).

## Abbreviations

AMPK: Activation of AMP-activated protein kinase
EDL: Extensor digitorum longus
eNOS: Endothelial nitric oxide synthase
FOXO1: Forkhead box protein O1
IGF: Insulin-like growth factor
IRS2: Insulin receptor substrate 2
MuRF: Muscle RING-finger protein
NF-κB: Nuclear Factor-κB p65
PGC: Peroxisome proliferator-activated receptor γ coactivator
SD: Sprague–Dawley
SDT: Spontaneously Diabetic Torii
S6K: Ribosomal protein S6 kinase beta-1
