# Supplementary material for: Mechanism for exercise-mediated prevention against muscle wasting on extensor digitorum longus muscle in Spontaneously Diabetic Torii fatty rats
Source: J Physiol Sci. 2023 Apr 4;73:5. doi: 10.1186/s12576-023-00865-5 (PMC10717411; doi:10.1186/s12576-023-00865-5)
Supplement: Supplementary file 1 — Additional file 1: Table S1 Muscle strength at 8-, 12-, and 16-week-old. Table S2 Muscle weight in 16-week-old SD and SDT fatty rats. Table S3 Cross-sectional area of type I and type IIb muscle fibers in 16-week-old SD and SDT fatty rats. Figure S1. Changes in body weight (a) and blood glucose levels (b) at 8-, 12-, and 16-week-old. SD-Cont group, n = 5; SD-Ex group, n = 5; SDT-Cont group, n = 6; and SDT-Ex group, n = 6. Values are presented as the mean ± standard error of the mean (SEM). *P < 0.05 and **P < 0.01 versus the SD group at the same age; # P < 0.05 and ## P < 0.01 versus the SD-Ex group at the same age; †P < 0.05 and ††P < 0.01 versus the same group at 8-week-old. These figures were created based on the results of reference No.18. Figure S2 Western blot analysis of p-Akt in EDL (a) and soleus (b) muscles, Akt in EDL (c) and soleus (d) muscles, and α-tubulin in EDL (e) and soleus (f) muscles on the same membrane in each group. Red lines represent the edge of each cut membrane. Red boxes show the regions of the original blots used in main figures. Figure S3 Western blot analysis of p-S6K in EDL (a) and soleus (b) muscles, S6K in EDL (c) and soleus (d) muscles, and α-tubulin in EDL (e) and soleus (f) muscles on the same membrane in each group. Red lines represent the edge of each cut membrane. Red boxes show the regions of the original blots used in main figures. Figure S4 Western blot analysis of p-Foxo1 in EDL (a) and soleus (b) muscles, Foxo1 in EDL (c) and soleus (d) muscles and α-tubulin in EDL (e) and soleus (f) muscles on the same membrane in each group. Red lines represent the edge of each cut membrane. Red boxes show the regions of the original blots used in main figures. Figure S5 Western blot analysis of p-NF-B in EDL (a) and soleus (b) muscles, NF-B in EDL (c) and soleus (d) muscles and α-tubulin in EDL (e) and soleus (f) muscles on the same membrane in each group. Red lines represent the edge of each cut membrane. Red boxes show the regions of [file 12576_2023_865_MOESM1_ESM.pdf]

Mechanism for exercise-mediated prevention against muscle wasting on extensor digitorum longus muscle in Spontaneously Diabetic Torii fatty rats

Running head: Exercise in SDT fatty rats

Hitoshi Kotake[1], Yuji Ogura[2], Shohei Yamada[1], Kazuho Inoue[3], Shiika Watanabe[1], Daisuke Ichikawa[1], Takeshi Sugaya[1], Keiichi Ohata[1], Yasunori Natsuki[4], Seiko Hoshino[3], Minoru Watanabe [5], Kenjiro Kimura[6], Yugo Shibagaki[1] and Atsuko Kamijo-Ikemori[1, 3, 5*]

1. Division of Nephrology and Hypertension, Department of Internal Medicine, St. Marianna University School of Medicine, Kanagawa, Japan.
2. Department of Physiology, St. Marianna University School of Medicine, Kanagawa, Japan.
3. Department of Anatomy, St. Marianna University School of Medicine, Kanagawa, Japan.
4. Institute for Ultrastructural Morphology, St. Marianna University School of Medicine, Kawasaki, Japan.
5. Institute for Animal Experimentation, St. Marianna University Graduate School of Medicine, Kanagawa, Japan.
6. JCHO Tokyo Takanawa Hospital, Tokyo, Japan.

Correspondence should be addressed to:

Atsuko Kamijo-Ikemori, M.D., Ph.D. *

Department of Anatomy, St. Marianna University School of Medicine,
Miyamae-Ku, Kawasaki 216-8511, Japan
Tel: +81-44-977-8111 ext. 3630, Fax: +81-44-976-7083
E-mail: a2kamijo@marianna-u.ac.jp

Supporting Information

Animals

All SDT fatty and SD rats used in the previous study were allowed free access to laboratory chow (CRF-2; Charles River Laboratories Japan, Yokohama, Japan) and water and were housed in the Institute for Animal Experimentation at St. Marianna University School of Medicine under a controlled temperature (24 °C) and 12-h light/dark cycle.

Muscle strength during an observational period

Muscle strength was measured every 4 weeks from ages 8 to 16 weeks using the forelimb grip test with a grip strength meter (MK-380CM/FM; Muromachi Kikai, Co., Ltd., Tokyo, Japan). The average of three measurements of muscle strength per animal per time point was used for the comparative analysis. The muscle strengths of all the rats were normalized to their body weight. The mean muscle strength was weaker in the SDT fatty group than in the SD group, but not significantly. Although no significant changes were found throughout the experimental period in each group, the muscle strength was significantly weaker in the SDT-Cont group than in the SD group at ages 12 and 16 weeks. In the SDT-Ex group, muscle strength was significantly greater than in the SDT-Cont group at 12- and 16-week-old, although it was significantly lower than in SD-Ex group at age 16 weeks (Table S1). These method and results were reported in detail in reference No.18.

Table S1 Muscle strength at 8-, 12-, and 16-week-old.

Muscle strength per body weight, N/kg	SD-Cont group, n = 5	SD-Ex group, n = 5	SDT-Cont group, n = 6	SDT-Ex group, n = 6
8-week-old	40.0 (2.8)	36.0 (4.1)	28.6 (1.2)	28.3 (2.8)
12-week-old	36.3 (1.6)	37.7 (2.8)	22.4 (1.1) $^{**,\ \#\#}$	31.5 (1.1) §§
16-week-old	42.5 (3.3)	50.3 (2.9)	27.5 (1.9) $^{**,\ \#\#}$	34.6 (2.0) $^{\#\#,\ \S}$

All data were expressed as the mean (SEM). $^{**}P < 0.01$ versus the SD-Cont group at the same age; $^{\#\#}P < 0.01$ versus the SD-Ex group at the same age; $^{\S}P < 0.05$ and $^{\S\S}P < 0.01$ versus the SDT-Cont group at the same age. This table was created based on the results of reference No.18.

Muscle weight in 16-week-old SD and SDT fatty rats

The soleus muscle and the extensor digitorum longus (EDL) muscle were removed after 16 hours of starvation at 16-week-old and their weight was measured. The muscle weights of all the rats were normalized to their body weight. Although the muscle weights of the soleus and EDL muscles were significantly lighter in the SDT fatty group than in the SD group, the muscle weight of the EDL muscle was significantly heavier in the SDT-Ex group than in the SDT-Cont group.

Table S2 Muscle weight in 16-week-old SD and SDT fatty rats

Muscle weight per body weight, g/kg	SD-Cont group, n = 5	SD-Ex group, n = 5	SDT-Cont group, n = 6	SDT-Ex group, n = 6
Soleus muscle	0.63 (0.02)	0.57 (0.03)	0.45 (0.01) $^{**, \#\#}$	0.47 (0.01) $^{**, \#\#}$
Extensor digitorum longus muscle	1.05 (0.02)	1.00 (0.03)	0.57 (0.01) $^{**, \#\#}$	0.61 (0.01) $^{**, \#\#, \S}$

All data were expressed as the mean (SEM). $^{**}P < 0.01$ versus the SD-Cont group at the same age; $^{\#\#}P < 0.01$ versus the SD-Ex group at the same age; § $P < 0.05$ versus the SDT-Cont group at the same age. This table was created based on the results of reference No.18.

Immunohistological analysis of muscle tissues in 16-week-old SD and SDT fatty rats

The frozen excised soleus and EDL muscle tissues were cut centrally along the width and embedded in Tissue-Tek O.C.T. Compound (Sakura Finetek Japan Co., Ltd., Tokyo, Japan). In double-color immunofluorescent staining, the cross-sectional areas of the specimens were observed and the diameter of each type I and IIb fiber was measured using 300 fibers in two visual fields at ×100 magnification per animal, by using an image analyzer (WinRoof). The cross-sectional areas of type I muscle fibers in the SDT fatty rats were significantly smaller than those in SD-Ex rats, but not in SD-Cont rats. While the cross-sectional areas of type IIb muscle fibers were significantly larger in the SD-Ex group than in the SD-Cont group, these were significantly smaller in the SDT-Cont group than in the SD group. In SDT-Ex group, although the cross-sectional areas of type IIb muscle fibers were significantly smaller than in the SD-Ex group, these were significantly larger than in the SDT-Cont group. These method and results were reported in detail in reference No.18.

Table S3 Cross-sectional area of type I and type IIb muscle fibers in 16-week-old SD and SDT fatty rats

Cross-sectional area of muscle fibers per body weight, arbitrary unit/kg	SD-Cont group, n = 5	SD-Ex group, n = 5	SDT-Cont group, n = 6	SDT-Ex group, n = 6
Type I muscle fibers	3299 (202)	3607 (164)	2651 (138) $^{\#}$	2824 (147) $^{\#}$
Type IIb muscle fibers	1838 (57)	2260 (77) *	1522 (70) $^{*, \#\#}$	1806 (87) $^{\#, \S}$

All data were expressed as the mean (SEM). $^{*}P < 0.05$ versus the SD-Cont group at the same age; $^{\#}P < 0.05$ and $^{\#\#}P < 0.01$ versus the SD-Ex group at the same age; $^{\S}P < 0.05$ versus the SDT-Cont group at the same age. This table was created based on the results of reference No.18.

Body weight and blood glucose levels during an observational period

Body weight and blood glucose levels were measured in all the rats every 4 weeks from ages 8 to 16 weeks. Body weight of all the SDT fatty rats was significantly heavier than that of all the SD rats at 8-week-old (Fig. S1a). While the body weights of SD-Ex group were significantly lighter than those of the SD-Cont group at 12- and 16-week-old, there were no significant differences between the SDT-Cont and SDT-Ex groups (Fig. S1a). Blood glucose levels were significantly higher in the SDT fatty group compared with the SD group and were similar between the SDT-Cont and SDT-Ex groups (Fig. S1b). These results were reported in reference No.18.

Figure S1

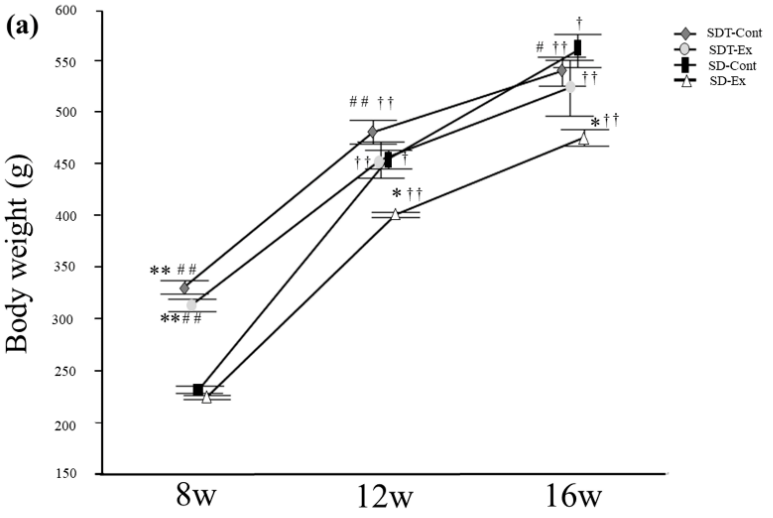

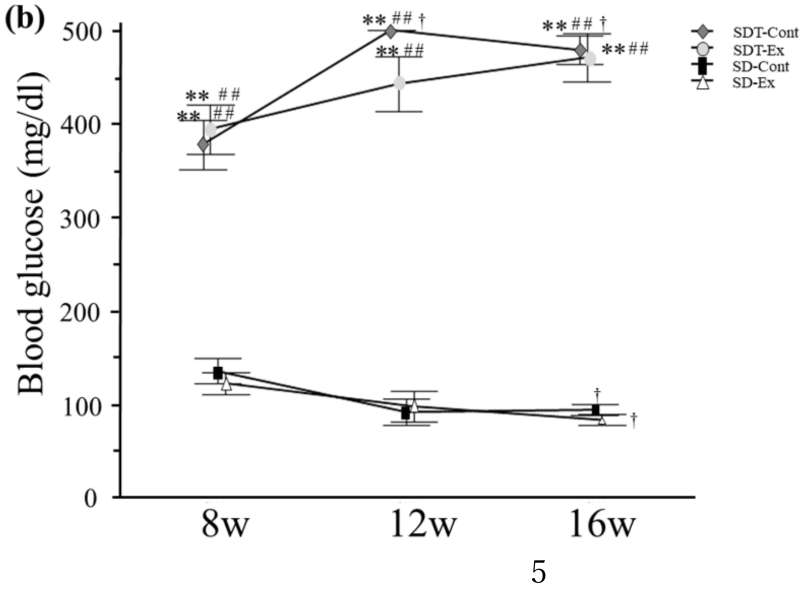

Figure S1 legend

Changes in body weight (a) and blood glucose levels (b) at 8-, 12-, and 16-week-old. SD-Cont group, n = 5; SD-Ex group, n = 5; SDT-Cont group, n = 6; and SDT-Ex group, n = 6. Values are presented as the mean ± standard error of the mean (SEM). $^{*}P < 0.05$ and $^{**}P < 0.01$ versus the SD group at the same age; $^{\#}P < 0.05$ and $^{\#\#}P < 0.01$ versus the SD-Ex group at the same age; $^{\dagger}P < 0.05$ and $^{\dagger\dagger}P < 0.01$ versus the same group at 8-week-old. These figures were created based on the results of reference No.18.

Entire images of western blotting

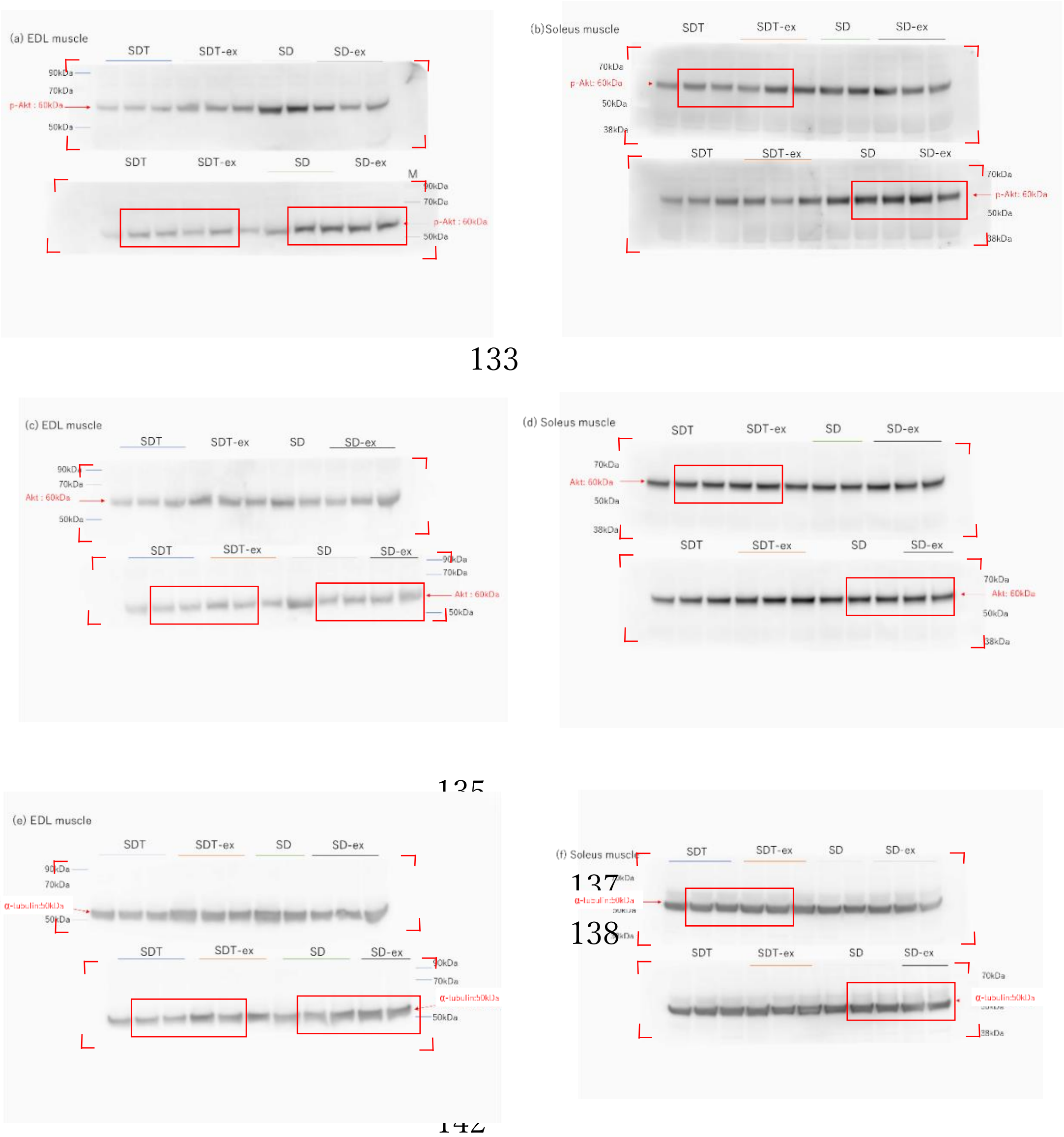

Figure S2 Western blot analysis of p-Akt in EDL (a) and soleus (b) muscles, Akt in EDL (c) and soleus (d) muscles, and α-tubulin in EDL (e) and soleus (f) muscles on the same membrane in each group. Red lines represent the edge of each cut membrane. Red boxes show the regions of the original blots used in main figures.

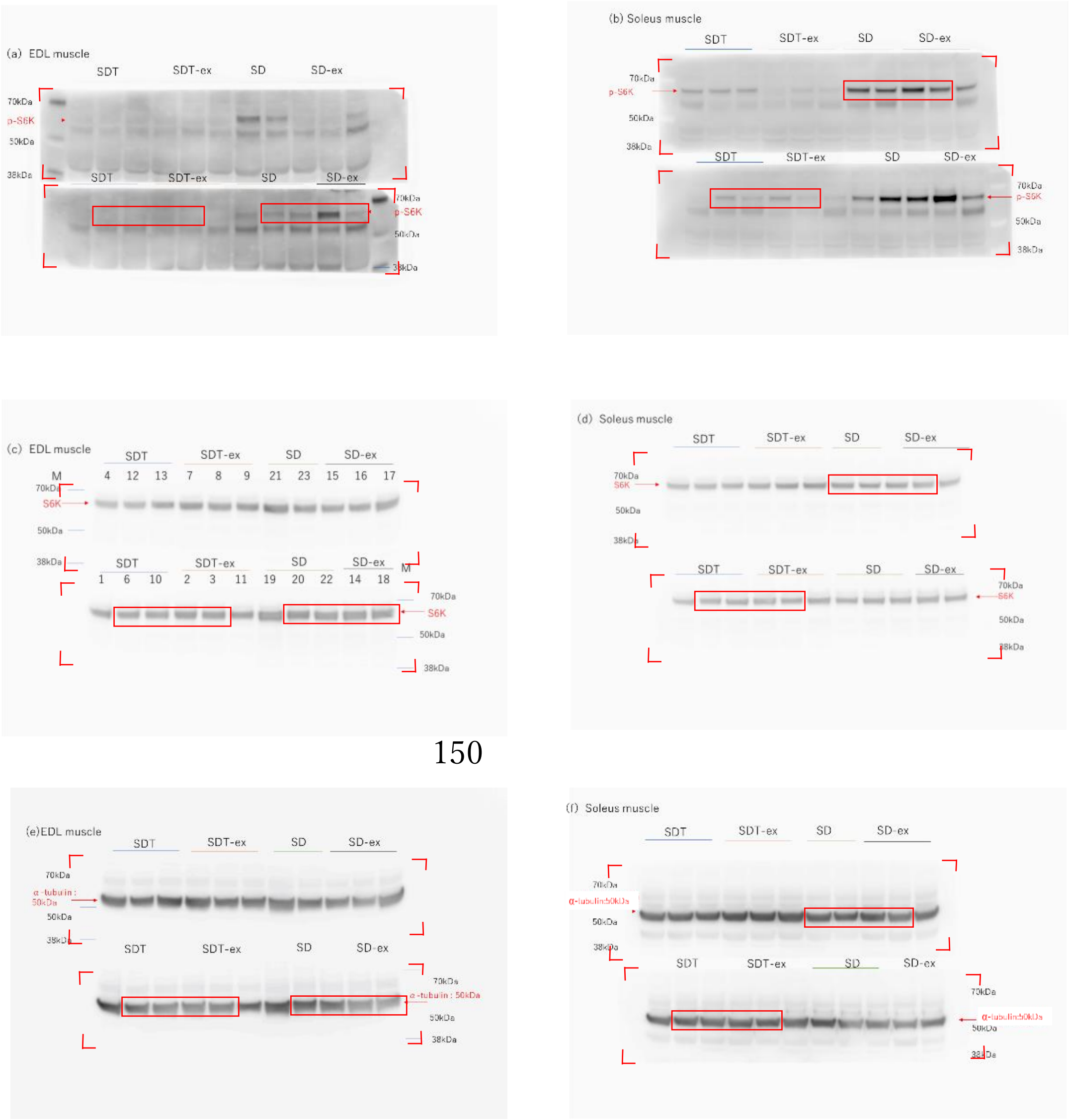

Figure S3 Western blot analysis of p-S6K in EDL (a) and soleus (b) muscles, S6K in EDL (c) and soleus (d) muscles, and α-tubulin in EDL (e) and soleus (f) muscles on the same membrane in each group. Red lines represent the edge of each cut membrane. Red boxes show the regions of the original blots used in main figures.

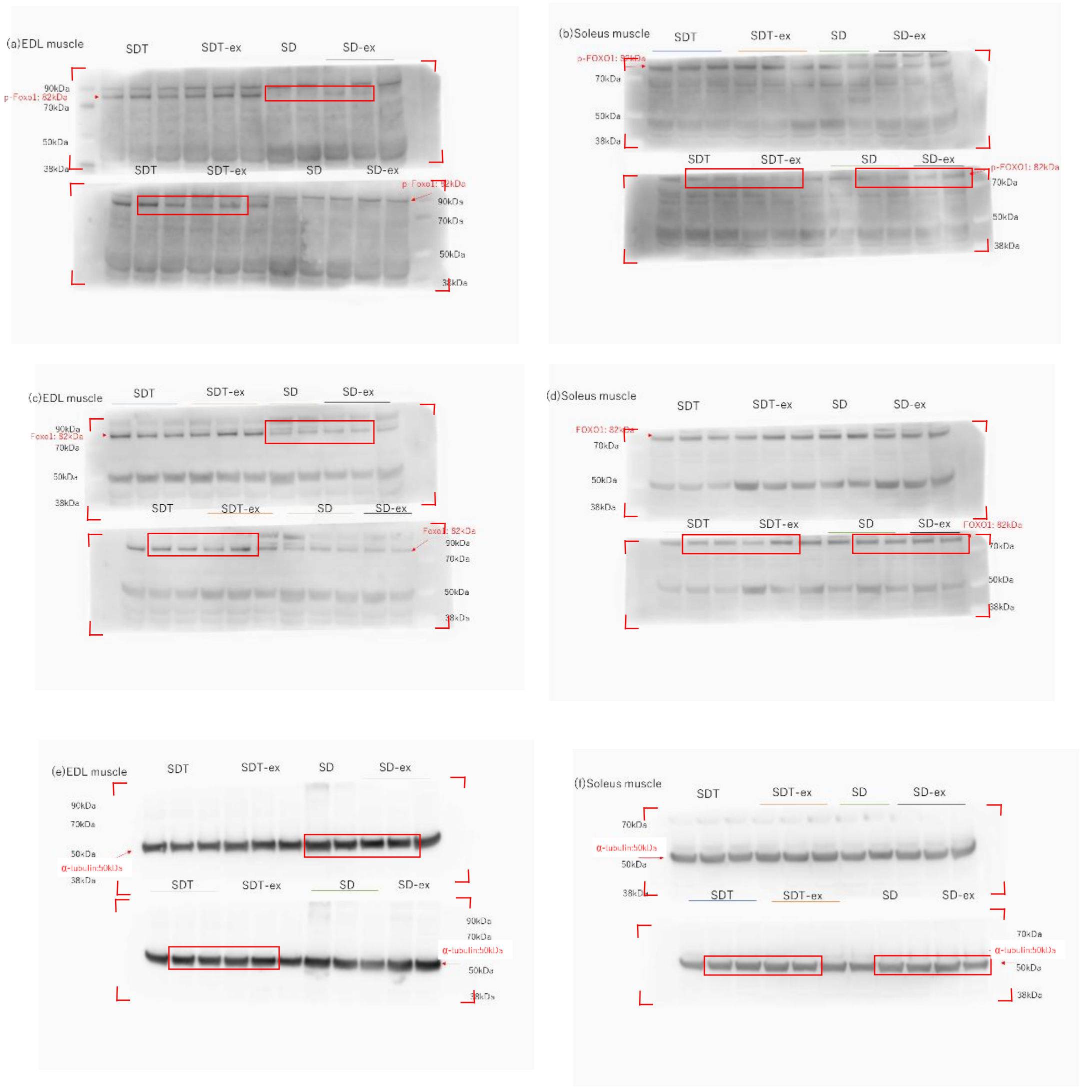

Figure S4 Western blot analysis of p-Foxo1 in EDL (a) and soleus (b) muscles, Foxo1 in EDL (c) and soleus (d) muscles and α-tubulin in EDL (e) and soleus (f) muscles on the same membrane in each group. Red lines represent the edge of each cut membrane. Red boxes show the regions of the original blots used in main figures.

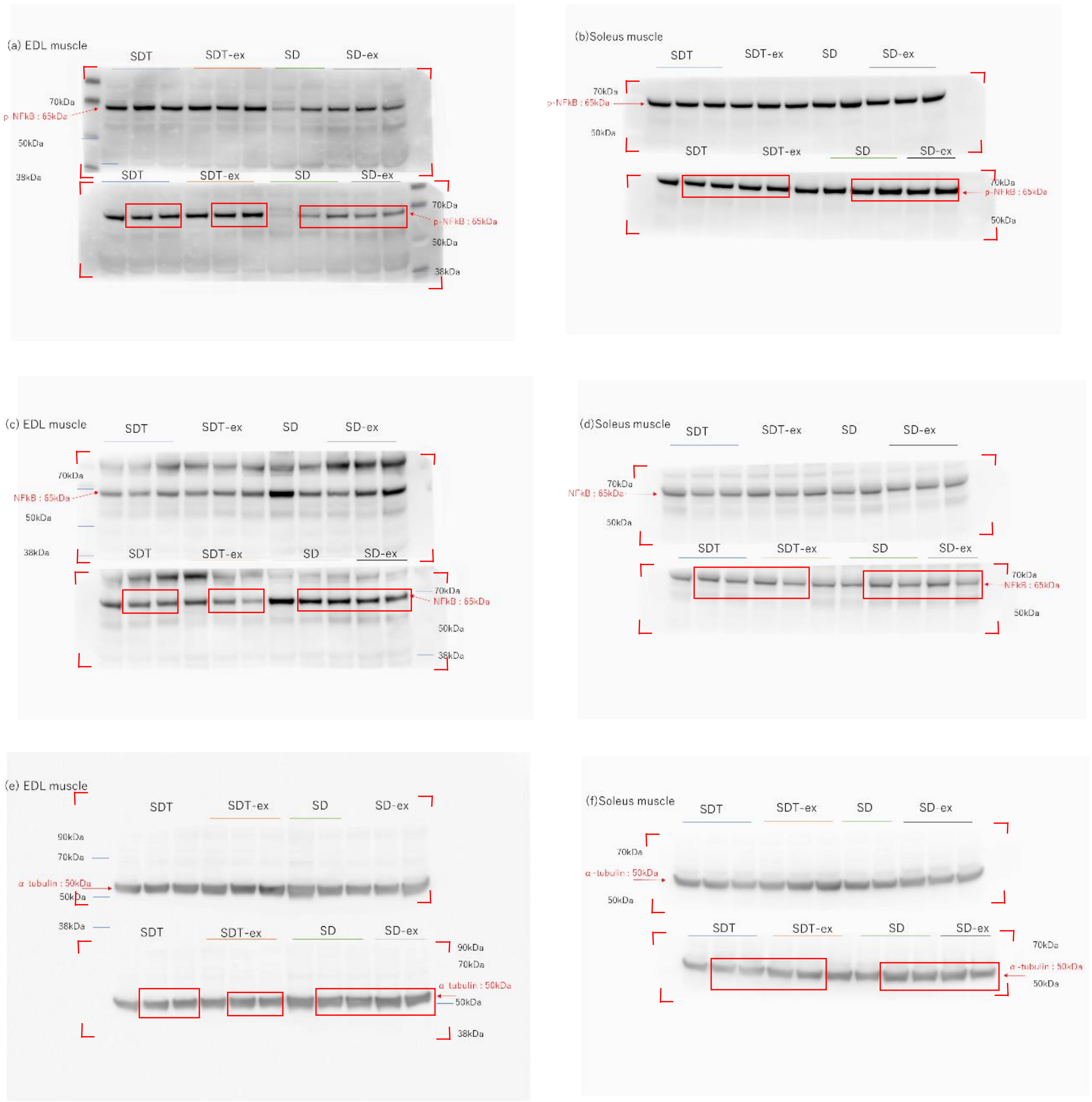

Figure S5 Western blot analysis of p-NF-κB in EDL (a) and soleus (b) muscles, NF-κB in EDL (c) and soleus (d) muscles and α-tubulin in EDL (e) and soleus (f) muscles on the same membrane in each group. Red lines represent the edge of each cut membrane. Red boxes show the regions of the original blots used in main figures.

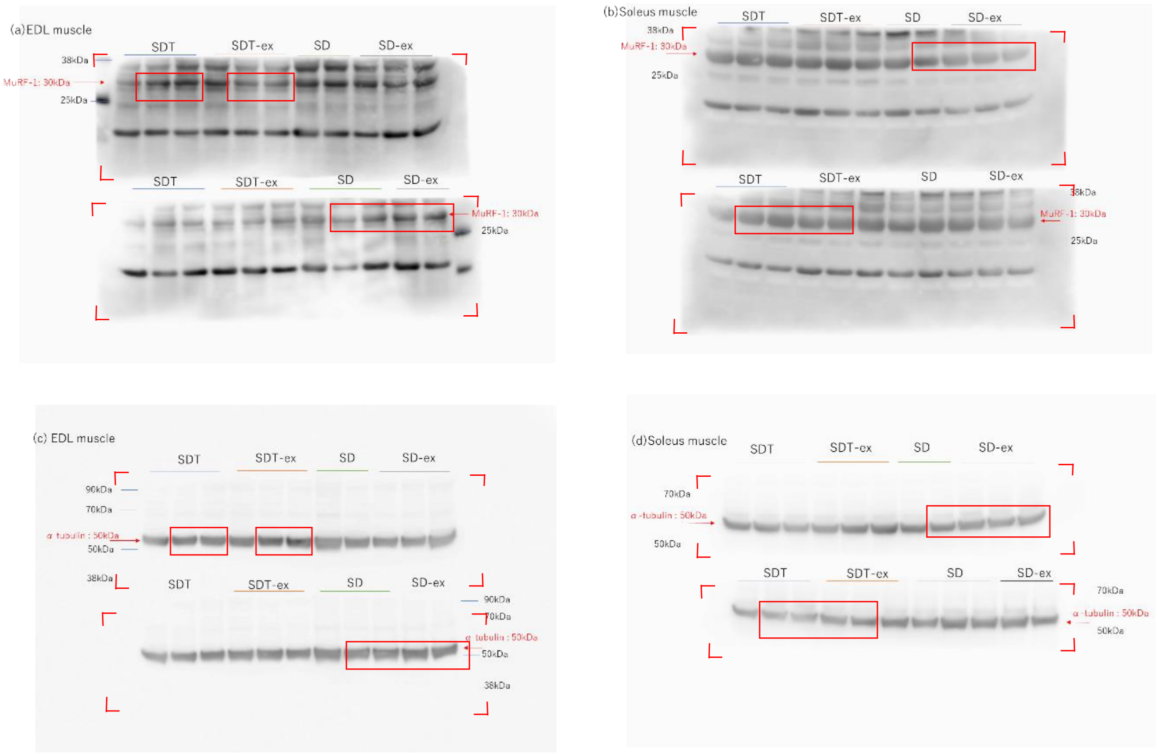

Figure S6 Western blot analysis of MuRF1 in EDL (a) and soleus (b) muscles, and α-tubulin in EDL (c) and soleus (d) muscles on the same membrane in each group. Red lines represent the edge of each cut membrane. Red boxes show the regions of the original blots used in main figures.

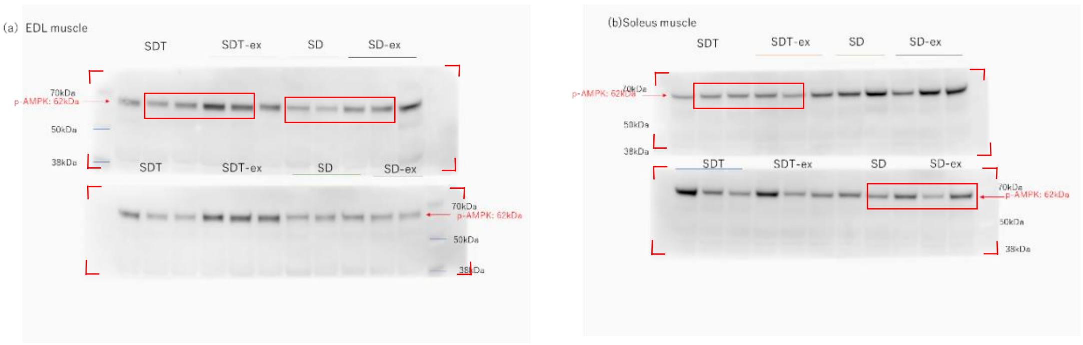

224

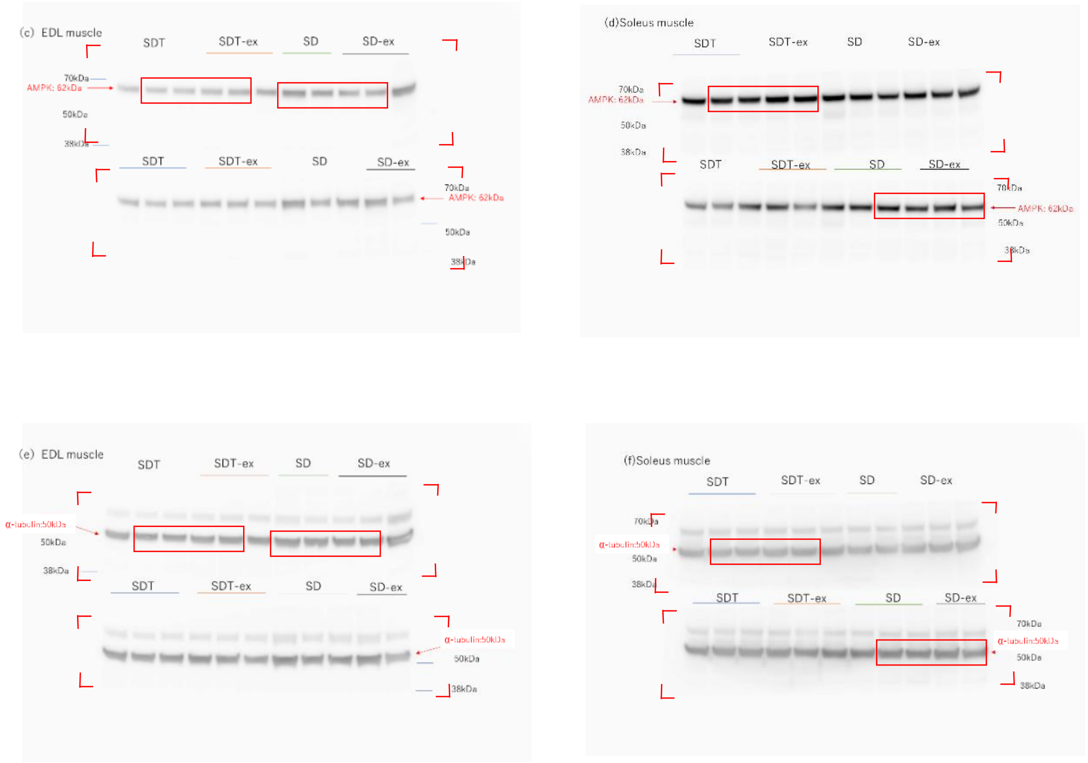

227

Figure S7 Western blot analysis of p-AMPK in EDL (a) and soleus (b) muscles, AMPK in EDL (c) and soleus (d) muscles, and α-tubulin in EDL (e) and soleus (f) muscles on the same membrane in each group. Red lines represent the edge of each cut membrane. Red boxes show the regions of the original blots used in main figures.

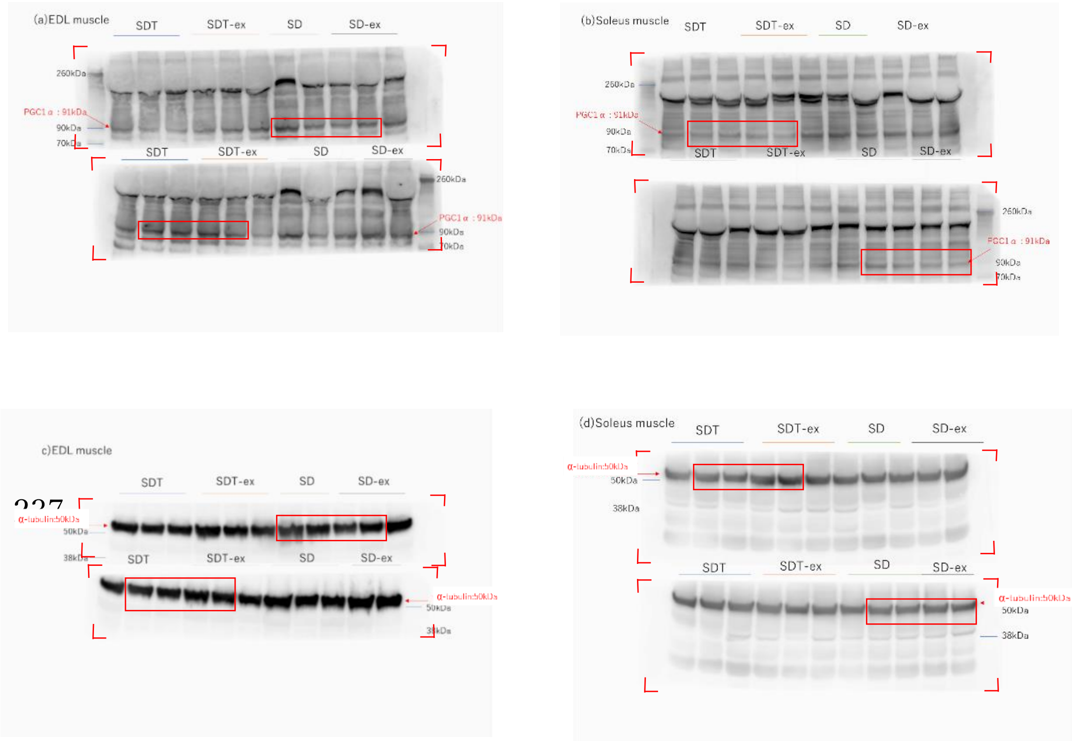

Figure S8 Western blot analysis of PGC1α in EDL (a) and soleus (b) muscles, and α-tubulin in EDL (c) and soleus (d) muscles on the same membrane in each group. The red lines represent the edge of each cut membrane. Red lines represent the edge of each cut membrane. Red boxes show the regions of the original blots used in main figures.

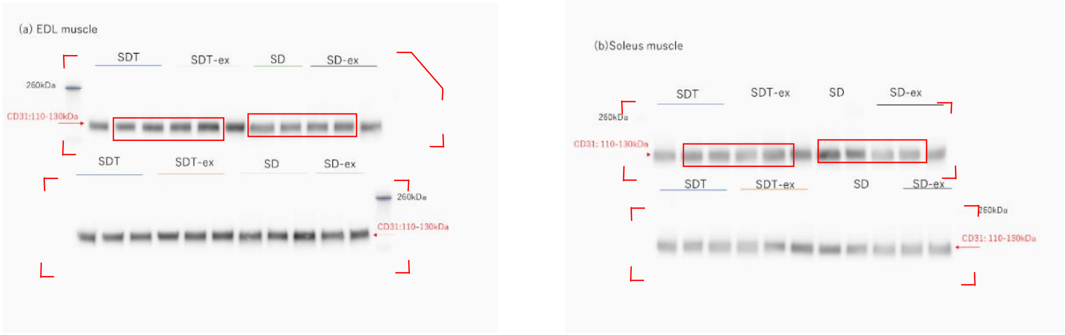

251

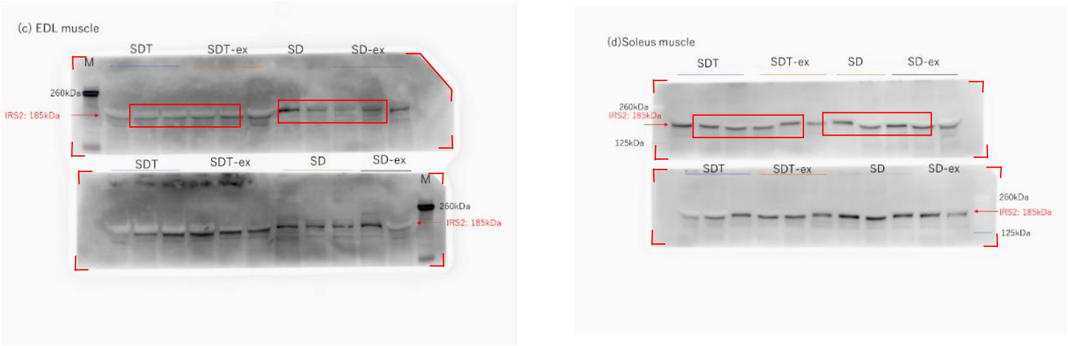

252

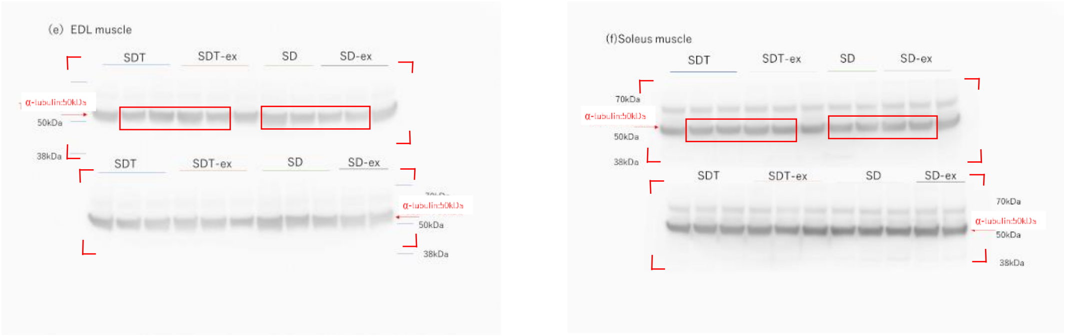

253

Figure S9 Western blot analysis of CD31 in EDL (a) and soleus (b) muscles, IRS2 in EDL (c) and soleus (d) muscles, and α-tubulin in EDL (e) and soleus (f) muscles on the same membrane in each group. Red lines represent the edge of each cut membrane. Red boxes show the regions of the original blots used in main figures.

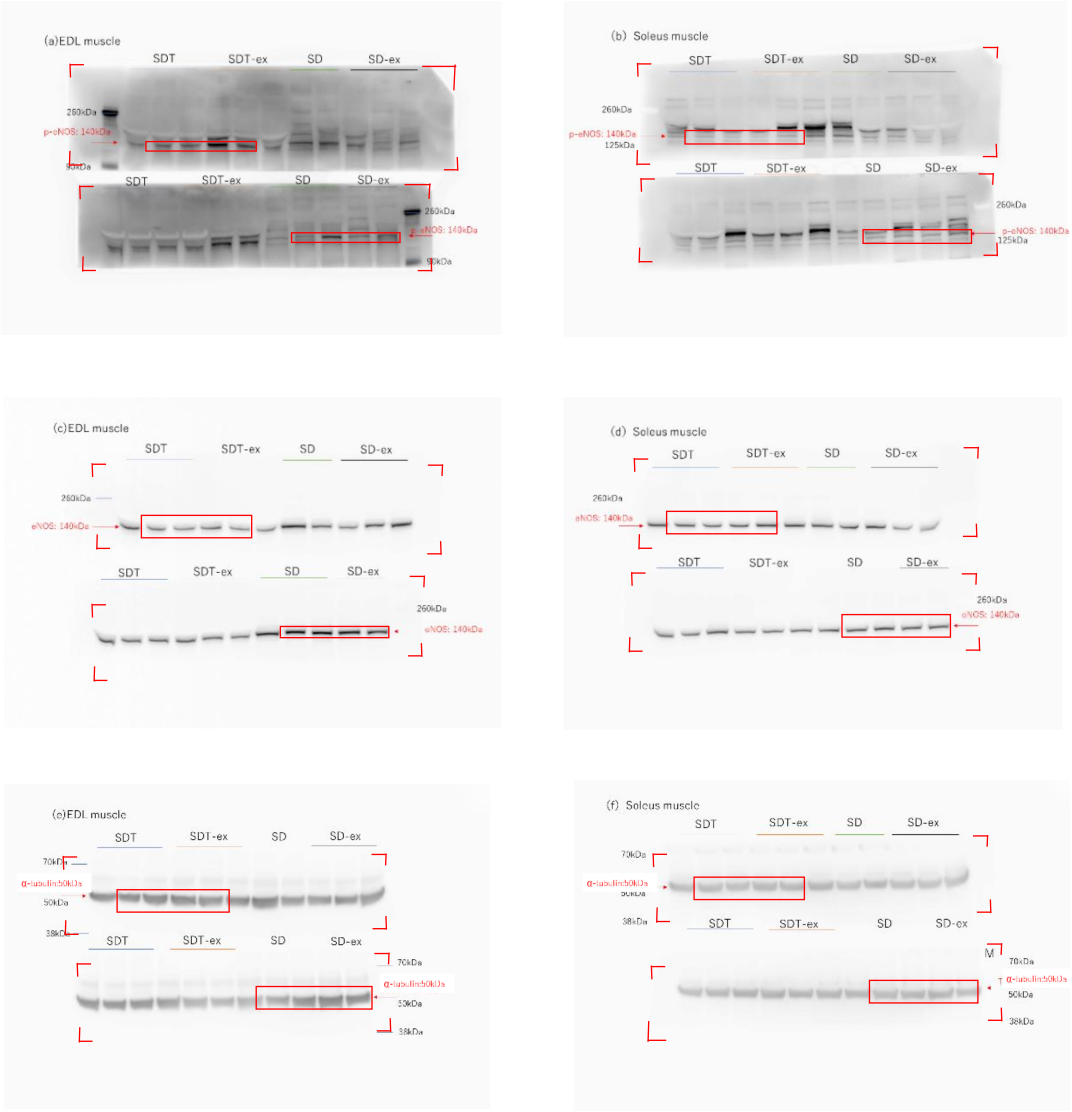

Figure S10 Western blot analysis of p-eNOS in EDL (a) and soleus (b) muscles, eNOS in EDL (c) and soleus (d) muscles, and α-tubulin in EDL (e) and soleus (f) muscles on the same membrane in each group. Red lines represent the edge of each cut membrane. Red boxes show the regions of the original blots used in main figures.
